# Identification of SNPs and InDels associated with berry size in table grapes integrating genetic and transcriptomic approaches

**DOI:** 10.1186/s12870-020-02564-4

**Published:** 2020-08-03

**Authors:** Claudia Muñoz-Espinoza, Alex Di Genova, Alicia Sánchez, José Correa, Alonso Espinoza, Claudio Meneses, Alejandro Maass, Ariel Orellana, Patricio Hinrichsen

**Affiliations:** 1 Instituto de Investigaciones Agropecuarias, INIA-La Platina, Santa Rosa 11610, Santiago, Chile; 2 Centro de Biotecnología Vegetal, Universidad Andrés Bello, Av. República 330, 3rd floor, Santiago, Chile; 3 Center for Mathematical Modeling (UMI2807-CNRS) and Department of Mathematical Engineering, Faculty of Mathematical and Physical Sciences, Universidad de Chile, Av. Blanco Encalada 2120, 7th floor, Santiago, Chile; 4 Center for Genome Regulation, Av. Blanco Encalada 2085, 3rd floor, Santiago, Chile

**Keywords:** *Vitis vinifera*, Berry size, SNP, InDel, RNA-Seq, Plant breeding, Marker-assisted selection

## Abstract

**Background:**

Berry size is considered as one of the main selection criteria in table grapes breeding programs, due to the consumer preferences. However, berry size is a complex quantitive trait under polygenic control, and its genetic determination of berry weight is not yet fully understood. The aim of this work was to perform marker discovery using a transcriptomic approach, in order to identify and characterize SNP and InDel markers associated with berry size in table grapes. We used an integrative analysis based on RNA-Seq, SNP/InDel search and validation on table grape segregants and varieties with different genetic backgrounds.

**Results:**

Thirty SNPs and eight InDels were identified using a transcriptomic approach (RNA-Seq). These markers were selected from SNP/InDel found among segregants from a Ruby x Sultanina population with contrasting phenotypes for berry size. The set of 38 SNP and InDel markers was distributed in eight chromosomes. Genotype-phenotype association analyses were performed using a set of 13 RxS segregants and 41 table grapes varieties with different genetic backgrounds during three seasons. The results showed several degrees of association of these markers with berry size (10.2 to 30.7%) as other berry-related traits such as length and width. The co-localization of SNP and /or InDel markers and previously reported QTLs and candidate genes associated with berry size were analysed.

**Conclusions:**

We identified a set of informative and transferable SNP and InDel markers associated with berry size. Our results suggest the suitability of SNPs and InDels as candidate markers for berry weight in seedless table grape breeding. The identification of genomic regions associated with berry weight in chromosomes 8, 15 and 17 was achieved with supporting evidence derived from a transcriptome experiment focused on SNP/InDel search, as well as from a QTL-linkage mapping approach. New regions possibly associated with berry weight in chromosomes 3, 6, 9 and 14 were identified.

## Background

Grapes (*Vitis vinifera* L.) is the main fruit crop of temperate regions, with a harvested area of 6.9 million of hectares in 2017 and 74.3 million tons of fruit produced throughout the world in the same period [1]. The grape is a long-lived liana adapted to a diverse range of climates [2–5]. It has a high level of genetic and phenotypic diversity, represented by the estimated 5000–10,000 existing cultivars of grapes [6, 7], including the whole genus *Vitis* with over 50 species [8–10].

Although high genetic diversity is an advantage for the success of breeding programs [11], it also represents a challenge. The characterization of the existing genotypes is it is very useful to optimize the breeding programs as well as to assist in the development of new varieties. However, juvenility of grapes delays phenotypic evaluation a few years, in particular when fruit characteristics are the main target. This represents a high cost in labor and land space dedicated to the segregant maintenance and evaluation. Therefore, the use of molecular tools providing phenotypic information at early stages of development for new lines can assist the selection (or discarding) of a subset of genotypes, and thus save resources and time.

Due to consumer preferences, the main selection criteria in table grape breeding include berry size and seedlessness (or a reduced seed production) [12], along with organoleptic traits such as flavor and aroma, among others. A strong correlation between berry size and seed content has been described, which is explained by the expression of a particular growth hormone family (gibberellins) in this organ [13–15]. Berry size is frequently handled in the field using exogenous applications of gibberellic acid (GA_3_), with different purposes in wine and table grapes varieties; in the former, the aim is to augment bunch loosening and aeration, while in the latter it is used mainly to increase berry size [16, 17], being especially important in seedless grape varieties to achieve commercial size.

Berry size is a polygenic quantitative trait. Numerous processes such as cell multiplication, cell wall modification, water and sugar transport probably define its expression [18–20], implying that numerous genes could contribute to explain berry size phenotypic variance. Despite its relatively high heritability and productive impact, the genetic determination of berry size is not yet fully understood [18, 19].

Several QTLs associated to berry size have been reported in linkage groups 1, 12 [21], 5, 13 [22], 8, 11, 17 [18], 15 [23], and 18 [24]. However, the low resolution of QTL analysis (Mbp scale) hinders their transferability as useful markers in practice [25].

The genes VvCEB1 and VvNAC26 were recently reported as major factors determining berry size. VvCEB1 is a bHLH transcription factor involved in the regulation of cell size in cv. Cabernet Sauvignon [26], while VvNAC26 was proposed as a factor determining berry size variation in a wide collection of grape genotypes [27]. Recently we reported and identified a group of candidate genes associated with berry size using transcriptomic data. The candidate genes encode for bHLH transcription factors, a GDSL esterase/lipase, a stilbene synthase, HSP17.9-D, among others [20]. Despite these findings, the genetic determinism and the molecular mechanisms behind berry development are complex and futher analysis and data are required to reveal their genetic architecture. Also, it is necessary to take into account the differential domestication process for table and wine grapes regarding berry size [28]. In genotypes being selected for the production of wine, the tendency was to smaller berries, in order to acummulate more anthocyanin pigments that are concentrated in the skin [29]. Wine *cepages* (in particular red ones) require certain degree of astringency, what is provided by the tannins present in seeds [28]. Thus, wine and table grapes diverged also in the growing response to the presence of gibberellins, provided mainly by seeds.

Berry size is an economically important trait for grape varieties [17]; it constitutes one of the main selection criteria for table grapes breeding programs among berry quality traits, based on tendency to select larger berries and clusters, in contrast to wine varieties harboring small seeded berries. Therefore, the identification of the genetic basis of berry size variation would provide invaluable knowledge about its genetic determinants [30], in search of molecular tools that could improve the breeding of the species by marker-assisted selection [20].

The development of molecular markers for early selection of seedlings is undoubtedly highly valuable [31, 32]. However, markers for fruit quality traits are still scarce [33]. Among the few examples, the marker for presence/absence of palatable seeds has been successfully applied in MAS [34], as well as the markers related to Muscat aroma [33, 35] and to fungus diseases, such as powdery mildew resistance [36–38], which are in use mostly in breeding programs intended for table grape varieties.

SNP/InDel represent a valuable source of genetic variability as drivers of new genes or allelic variants, which might be selected by natural or artificial ways when resultant phenotypes exhibit advantageous traits [28]. Marker search based on transcriptome sequencing using high-throughput sequencing has led to rapid discovery of polymorphisms focused on coding regions, avoiding highly repetitive genome regions. It has also become a powerful tool for the detection of causal variants/mutations [39–42]. Despite the large efforts and current wealth of information from transcriptome sequencing data (RNA-seq), its application for the identification of molecular markers such as SSR, SNP and InDel is still limited [43]. However, some few examples have been reported [42–44].

The aim of this study was to perform marker discovery using a transcriptomic approach, in order to identify and characterize SNP and InDel markers associated with berry size in table grape. For this we used a ‘Ruby seedless’ x ‘Sultanina’ (RxS) progeny (*N* = 139), a population containing segregants with contrasting phenotypes for berry size. Among the hundreds of markers initially identified, 30 SNPs and eight InDels were selected and experimentally validated in (RxS) segregants, in table grape varieties and in representative genotypes of a *V. vinifera* core collection.

## Results

### Global identification of SNPs and InDels related to berry size in the *V. vinifera* genome

A global search of single nucleotide polymorphisms (SNPs) and insertion/deletion (InDels) related to berry size was performed, using available data from an RNA-Seq experiment based on 14 samples collected at fruit setting and 6–8 mm berry diameter stages, as previously described [20] (Additional file 1: Table S1).

**Fig. 1:**
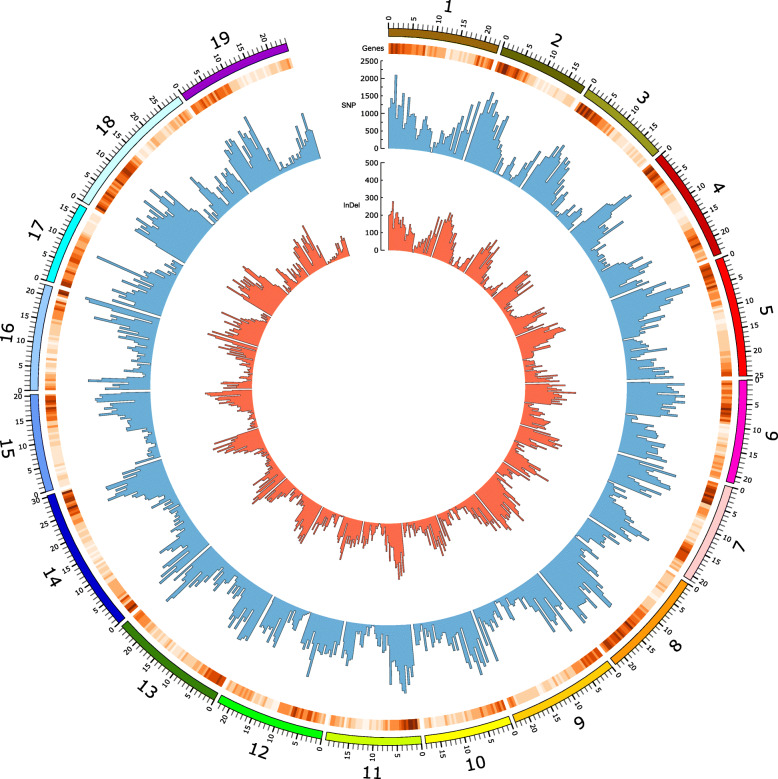
Distribution of SNP and InDel polymorphisms along the 19 chromosomes of *Vitis vinifera* (PN40024). The histograms in blue and red represent the number of SNPs and InDels per 0.5 Mbp bin, respectively; the heatmap represents the gene density estimated per 0.5 Mbp bin

The SNP/InDel calling was performed following the best practices recommended by the GATK software [45] and previously aligned to sequences of the *Vitis vinifera* reference genome using Tophat [46] (Additional file 2: Figure S1). A total of 771,118 SNPs and 100,205 short InDels were identified. Of the InDels, 55,989 corresponded to insertions and 44,216 to deletions (Fig. 1). The SNPs were rather homogeneously distributed along *V. vinifera* chromosomes (chr); the largest number was found in chr 18 (57,443) and the lowest in chr 17 (26,455), with an average of 37,709 SNPs per chromosome (Additional file 3: Figure S2A). Many of them were located in intronic regions (35.7%) of the reference genome, followed by exonic regions (14.7%), intergenic (14.3%), downstream (16.3%) and upstream (10.3%) (Additional file 4: Figure S3). There were 7.2% non-synonymous coding and 8.6% intragenic SNPs. The substitution rate between non-synonymus and synonymous was 0.98 in exonic regions. A total of 488,517 transitions and 284,341 transversions were observed, corresponding to 63 and 37%, respectively, with a transition/transversion ratio (Ts/Tv) of 1.7. The largest number of InDels was also found in chr 18 (7809) and the lowest on chr 10 (3251), with an average of 5095 InDels per chromosome (Additional file 3: Figure S2B). InDels were located frequently in intron regions (34.4%), following by downstream (16%), intergenic (11.6%), upstream (10.4%), and 8.5% were found in exon regions (Additional file 5: Figure S4). SNP and InDel density varied among chromosomes; however, the highest density was observed for both polymorphisms in chr 8 (2.15 SNPs per Kb, 0.32 InDels per Kb), while the lowest density for both was observed in chr 9 (1.32 SNPs per Kb, 0.18 InDels per Kb) (Additional file 6: Figure S5).

### In silico selection of SNPs and InDels associated to berry size

In order to select putative polymorphisms associated to berry size, we defined a set of criteria that candidate makers should fulfill (See methods section). Missing genotype data (e.g., the absence of reads for the identified polymorphisms) represented the most significant filter, corresponding to 84.5% in the case of SNPs and 77% in the case of InDels (Additional file 2: Figure S1). After this filtering, 41,034 SNPs and 7446 InDels were obtained. The remaining SNPs and InDels were selected using a fixation index (F_st_) of 1 [47], which implies that the allele frequency for each SNP or InDel of the same phenotypic class in ‘large berry’ (LB) segregants differed from those observed in ‘small berry’ (SB) segregants. F_ST_ analysis selected 382 SNPs and 16 InDels associated to berry size. Putative SNPs were mainly distributed in five chromosomes: 17 (37%), 9 (24%), 15 (12%), 6 (9%) and 8 (7%). The putative InDels were located mainly in chr 6 and 9 (25% each) and chr 11 (12.5%).

Functional anntotation was performed using snpEff software [48], according to the *V. vinifera* reference genome (PN40024) (Additional file 2: Figure S1). In addition, a functional enrichment analysis (Gene Ontology) was performed to determine main over-represented biological processes in the 232 genes where the 382 SNPs were located. We used the ShinyGO platform [49, 50] (Additional file 7: Figure S6). Our results showed that GO categories for biosynthetic process, response to stimulus, developmental process, localization and methylation were over-represented in the candidate gene set (*p* < 0.01) (Additional file 7: Figure S6).

Subsequently, 177 putative SNPs were selected due to their effects on CDS, 84 of them as ‘non-synonymous coding’ and 93 as intragenics (snpEff software, see methods). InDels were analyzed for frameshift, intragenics, intronics, as well as ‘*splice site donor’* and ‘*splice site acceptor*’. After the depth coverage analysis, defined as the total number of reads overlapping a given genomic position [51], 68 SNPs and 12 InDels were selected to design specific primers to perform their validation using High Resolution Melting Analysis (qPCR-HRM).

Therefore, there were experimental steps that caused the reduction of the markers to be evaluated, including sequences where primers and PCR products were not possible to be obtained.

In summary, primers were obtained for 45 SNPs and 12 InDels. SNP/Indel primers were discarded considering: 1) no amplification products were observed, or 2) ambiguous or not discriminative HRM/melting curves were obtained. Subsequently, sets of 34 and 10 primers were selected, respectively, for SNPs and InDels to be confirmed by Sanger sequencing. From them, 30 SNPs and eight InDelS were confirmed, representing a validation rate of 88% (30/34) and 80% (8/10), respectively.

### Experimental validation of SNP and InDel candidates associated with berry size by qPCR-HRM

To confirm and validate SNPs and InDels associated with berry size by qPCR-HRM, primers were designed for 30 SNPs and eight InDels, respectively (Additional file 8: Table S2, and Additional file 9: Table S3). SNP-calling quality indexes varied between 3899 and 110,535. Primer pairs amplifying fragments among 79 to 183 bp were selected, optimizing HRM and melting curves in order to have confident results.

Then polymorphisms were evaluated in a set of six seedless RxS segregants with contrasting phenotypes for berry weight, plus the parents. An equal number of transitions and transversions were validated; the most frequent changes were T- > C/C- > T and A- > C/C- > A, corresponding to nine and six SNPs, respectively.

Segregation patterns of parentals were ‘abxaa’ and ‘abxab’, indicating in the first case that each parental grouped with small berry (SB) or large berry segregant (LB) groups, according with [20]; in the case of the latter, both parents were heterozygous for the polymorphism and grouped with the SB or LB segregant sets.

**Fig. 2:**
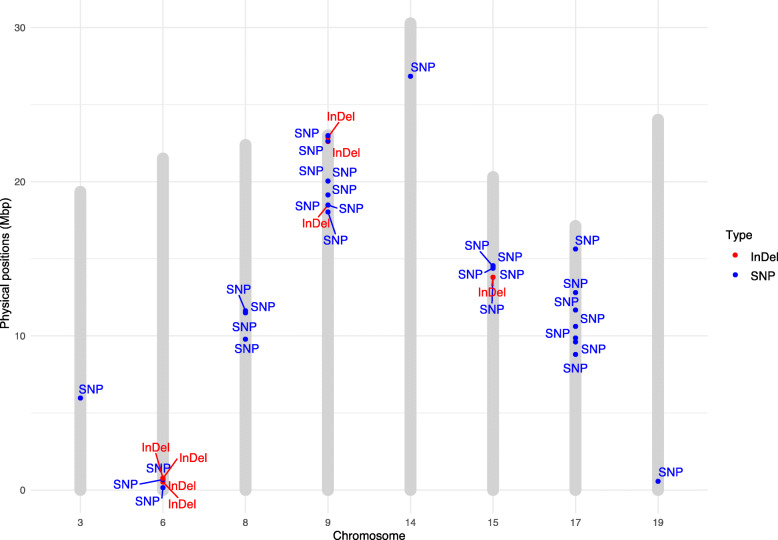
Distribution of 38 candidate polymorphisms for berry size, 30 SNPs plus eight InDels in *V. vinifera* chromosomes. Markers were located in 32 genes distributed in eight chromosomes

The 38 polymorphisms were distributed in eight chromosomes: 3, 6, 8, 9, 14, 15, 17 and 19, but a large percentage (82%) were located in chr 6, 9, 15 and 17 (Tables 1 and 2, Fig. 2). Co-localization of SNPs and InDels was observed at chromosomes 6, 9 and 15, suggesting a higher possibility of harboring genes related to berry weight (Fig. 2). In fact, 11 polymorphisms were located at chr 9 (29%), and seven at chr 6.

**Table 1:** Summary of 30 SNPs with association to berry fresh weight (BFW), derived from transcriptomic approach based on seedless segregants exhibiting contrasting phenotypes for berry weight. All of them were validated in RxS segregants and grapevine varieties as well

SNP_ID	Chr	Ref	Chg	Effect	Gene_ID	Description
TSRNASNPS120468689	3	G	A	Non-synonymous coding	Vitvi03g00541	Predicted Ca2 + −dependent phospholipid-binding protein
TSRNASNPS120570749	6	C	T	Non-synonymous coding	Vitvi06g00013	Unknown Protein Function
TSRNASNPS120571906	6	G	A	Intragenic	Vitvi06g00053	Importin-5
TSRNASNPS120572210	6	C	T	Non-synonymous coding	Vitvi06g00063	Pheophorbide a oxygenase chloroplastic
TSRNASNPS120668018	8	T	C	Non-synonymous coding	Vitvi08g00791	emb|CAB82287.1| putative protein
TSRNASNPS120671217	8	G	T	Intragenic	Vitvi08g00916	Unknown Protein Function
TSRNASNPS120671218	8	A	C	Intragenic	Vitvi08g00916	Unknown Protein Function
TSRNASNPS120671409	8	A	G	Non-synonymous coding	Vitvi08g00930	4-alpha-glucanotransferase
TSRNASNP120728591	9	C	T	Intragenic	Vitvi09g01229	Anthranilate N-benzoyltransferase protein 2
TSRNASNP120729221	9	T	C	Non-synonymous coding	Vitvi09g01251	Probable serine incorporator
TSRNASNP120729235	9	T	C	Non-synonymous coding	Vitvi09g01251	Probable serine incorporator
TSRNASNPS120730012	9	T	C	Non-synonymous coding	Vitvi09g01280	Unknown protein DS12 from 2D-PAGE of leaf chloroplastic
TSRNASNP120731088	9	A	C	Non-synonymous coding	Vitvi09g01317	Unknown Protein Function
TSRNASNP120731107	9	C	G	Non-synonymous coding	Vitvi09g01317	Unknown Protein Function
TSRNASNPS120734745	9	C	T	Non-synonymous coding	Vitvi09g01459	Myb family transcription factor APL
TSRNASNP120735535	9	C	G	Non-synonymous coding	Vitvi09g01484	Ubiquitin-like protein regulator of apoptosis
TSRNASNPS120185697	14	G	T	Intragenic	Vitvi14g01672	gb|ACG39074.1| lysine ketoglutarate reductase trans-splicing related 1
TSRNASNPS120206217	15	A	T	Non-synonymous coding	Vitvi15g00647	Beta-fructofuranosidase (invertase)
TSRNASNPS120206366	15	C	T	Intragenic	Vitvi15g00679	Phosphatidate cytidylyltransferase
TSRNASNPS120206375	15	A	G	Intragenic	VviHAM2	Scarecrow-like protein 6
TSRNASNPS120206839	15	T	A	Intragenic	Vitvi15g00690	Transketolase 10
TSRNASNPS120206984	15	A	G	Intragenic	Vitvi17g00727	Serine/threonine-protein kinase AtPK2/AtPK19
TSRNASNPS120273500	17	C	G	Non-synonymous coding	Vitvi17g00758	Unknown Protein Function
TSRNASNPS120275548	17	C	G	Non-synonymous coding	Vitvi17g00817	Peroxisomal membrane anchor protein (peroxin)
TSRNASNPS120275823	17	A	C	Non-synonymous coding	Vitvi17g00834	Predicted E3 ubiquitin ligase
TSRNASNPS120277213	17	C	A	Intragenic	Vitvi17g00894	Histone deacetylase 6
TSRNASNPS120278360	17	G	A	Intragenic	Vitvi17g00977	Probable pectate lyase 5
TSRNASNPS120279487	17	A	C	Intragenic	Vitvi17g01598	Glyceraldehyde-3-phosphate dehydrogenase cytosolic
TSRNASNPS120280865	17	C	A	Intragenic	Vitvi17g01207	Transcription factor BTF3 homolog 4
TSRNASNPS120346601	19	T	G	Intragenic	Vitvi19g00043	Cytosolic sorting protein GGA2/TOM1

**Table 2:** Summary of eight InDels with association to berry fresh weight (BFW), derived from transcriptomic approach based on seedless segregants exhibiting contrasting phenotypes for berry weight. All of them were validated in RxS segregants and grapevine varieties as well

InDel_ID	Chr	Change type	Effect	Gene_ID	Description
TSRNAINDELS120073669	6	DEL	Intragenic	Vitvi06g01581	Holocarboxylase synthetase
TSRNAINDELS120073728	6	DEL	Intragenic	Vitvi06g00050	Mitochondrial 2-oxoglutarate/malate carrier protein
TSRNAINDELS120073761	6	DEL	Intron	VviMYB5B	Transcription repressor MYB5
TSRNAINDELS120073788	6	DEL	Intragenic	Vitvi06g00063	Pheophorbide a oxygenase chloroplastic
TSRNAINDELS120095050	9	INS	Intragenic	Vitvi09g01251	Probable serine incorporator
TSRNAINDELS120095636	9	DEL	Intragenic	Vitvi09g01457	YTH domain family protein 2
TSRNAINDELS120095711	9	DEL	Frame_shift	Vitvi09g01486	Unknown Protein Function
TSRNAINDELS120025818	15	INS	Downstream	Vitvi15g00647	Beta-fructofuranosidase (invertase)

This group of 38 markers was linked to 32 genes, 27 genes harboring SNPs and eight genes showing InDels (Tables 1 and 2). Interestingly, two genes presented SNPs and InDels in their sequences, one coding for a probable serine incorporator (Vitvi09g01251, two SNPs/one InDel), and a gene coding for chloroplastic pheophorbide oxygenase (Vitvi06g00063, one SNP/one InDel); the latter gene has been related to plant senescence [52].

The group of 32 genes presenting polymorphisms associated to berry weight is a diverse group (Tables 1 and 2), including genes coding for histone deacetylase 6 (Vitvi17g00894), reported as a chromatin remodelling which contributes to transcriptional repression [53]. In addition, five genes coding for proteins with unknown function, three for transcription factors corresponding to the Myb family, a BTF3 homolog 4 and repressor MYB5 were identified. None of these genes showed significantly different expression when comparing large and small berry genotypes at different berry development times [20]. GO categories biosynthetic process and localization were over-represented in this set of genes (*p* < 0.05) (Additional file 10: Figure S7).

### Genotype-phenotype association analysis of SNPs/InDels for berry weight

The results were analyzed using a global linear model (GLM) [54], to determine if there is a statistical association of phenotypic data regarding berry weight with the information provided by the candidate markers. Individuals from the RxS progeny showed significant associations for SNP and InDel markers located in chr 6, 8, 15 and 17 with berry fresh weight (BFW) (*p* < 0.05) (Table 3a). A group of six candidate SNPs located in chromosome 17 encompassing a region of 6.8 Mbp exhibited high association with berry weight (Table 3a).

**Table 3:** Genotype-phenotype association analysis results including markers that showed significant associations with berry fresh weight (BFW) (A), bunch weight (BWe) (B), berry length (BL) (C), berry width (BWi) (D) and berry volume (BV) (E), as determined in a set 13 RxS segregants with contrasting phenotypes for berry size. *P*-values of associations and variance explained by each SNP/InDel marker (R^2^) are indicated for the GLM models obtained for seasons 2010, 2011 and 2012. Statistically significant associations are highlighted with an asterisk (*p* < 0.05)

A.							
		**BFW 2010**		**BFW 2011**		**BFW 2012**	
**Marker**	**Chr**	***p*-value**	**R**^**2**^**(%)**	***p*-value**	**R**^**2**^**(%)**	***p*-value**	**R**^**2**^**(%)**
TSRNAINDELS120073669	6	0.0173*	41.6	0.1572	18.9	0.0365*	34
TSRNAINDELS120073728	6	0.0419*	32.5	0.3932	7.4	0.0925	23.6
TSRNAINDELS120073761	6	0.0419*	32.5	0.3932	7.4	0.0925	23.6
TSRNAINDELS120073788	6	0.0419*	32.5	0.3932	7.4	0.0925	23.6
TSRNASNP120668018	8	0.2449	12.1	0.0073*	12.1	0.1162	12.1
TSRNASNP120671217	8	0.2449	12.1	0.0073*	12.1	0.1162	12.1
TSRNASNP120671218	8	0.2449	12.1	0,0073*	12.1	0.1162	12.1
TSRNASNP120671409	8	0.2449	12.1	0,0073*	12.1	0.1162	12.1
TSRNASNP120206984	15	0.0339*	49.2	0.2812	49.2	0.1126	49.2
TSRNASNP120273500	17	0.0019*	59.9	0.0191*	59.9	0.0005*	59.9
TSRNASNP120275823	17	0.0019*	59.9	0.0191*	59.9	0.0005*	59.9
TSRNASNP120277213	17	0.0019*	59.9	0.0191*	59.9	0.0005*	59.9
TSRNASNP120278360	17	0.0019*	59.9	0.0191*	59.9	0.0005*	59.9
TSRNASNP120279487	17	0.0019*	59.9	0.0191*	59.9	0.0005*	59.9
TSRNASNP120280865	17	0.0019*	59.9	0.0191*	59.9	0.0005*	59.9
TSRNASNP120275548	17	0.0134*	44.0	0.1049	44.0	0.0108*	44.0
B.							
		**BWe 2010**		**BWe 2011**		**BWe 2012**	
**Marker**	**Chr**	***p*-value**	**R**^**2**^**(%)**	***p*-value**	**R**^**2**^**(%)**	***p*-value**	**R**^**2**^**(%)**
TSRNASNP120273500	17	0.0430*	32.2	0.2441	13.3	0.0010*	67.8
TSRNASNP120275823	17	0.0430*	32.2	0.2441	13.3	0.0010*	67.8
TSRNASNP120277213	17	0.0430*	32.2	0.2441	13.3	0.0010*	67.8
TSRNASNP120278360	17	0,0430*	32.2	0.2441	13.3	0.0010*	67.8
TSRNASNP120279487	17	0.0430*	32.2	0.2441	13.3	0.0010*	67.8
TSRNASNP120280865	17	0.0430*	32.2	0.2441	13.3	0.0010*	67.8
TSRNASNP120275548	17	0.09226	23.6	0.1669	18.2	0.0025*	61.7
C.							
		**BL 2010**		**BL 2011**		**BL 2012**	
**Marker**	**Chr**	***p*-value**	**R**^**2**^**(%)**	***p*-value**	**R**^**2**^**(%)**	***p*-value**	**R**^**2**^**(%)**
TSRNAINDELS120073669	6	0.0463*	31.4	0.0334*	34.9	0.0589	28.8
TSRNASNP120668018	8	0.0952	23.2	0.0273*	37.1	0.3045	9.5
TSRNASNP120671217	8	0.0952	23.2	0.0273*	37.1	0,3045	9.5
TSRNASNP120671218	8	0.0952	23.2	0.0273*	37.1	0.3045	9.5
TSRNASNP120671409	8	0.0952	23.2	0.0273*	37.1	0.3045	9.5
TSRNAINDELS120025818	15	0.2293	25.5	0.3628	0.18	0.0312*	50.0
TSRNASNP120206984	15	02293	25.5	0.3628	0.18	0.0312*	50.0
TSRNASNP120273500	17	0.0083*	48.3	0.0140*	43.6	0.0059*	51.2
TSRNASNP120275823	17	0.0083*	48.3	0.0140*	43.6	0.0059*	51.2
TSRNASNP120277213	17	0.0083*	48.3	0.0140*	43.6	0.0059*	51.2
TSRNASNP120278360	17	0.0083*	48.3	0.0140*	43.6	0.0059*	51.2
TSRNASNP120279487	17	0.0083*	48.3	0.0140*	43.6	0.0059*	51.2
TSRNASNP120280865	17	0.0083*	48.3	0.0140*	43.6	0.0059*	51.2
TSRNASNP120275548	17	0.0738	26.2	0.0471*	31.2	0.0153*	42.8
D.							
		**BWi 2010**		**BWi 2011**		**BWi 2012**	
**Marker**	**Chr**	***p*-value**	**R**^**2**^**(%)**	***p*-value**	**R**^**2**^**(%)**	***p*-value**	**R**^**2**^**(%)**
TSRNAINDELS120073669	6	0.0223*	39.1	0.0104*	46.4	0.0214*	39.5
TSRNASNP120728591	9	0.0383*	33,5	0.0434*	32.1	0.0652	27.7
TSRNAINDELS120095050	9	0.0383*	33,5	0.0434*	32.1	0.0652	27.7
TSRNASNP120729221	9	0.0383*	33,5	0.0434*	32.1	0.0652	27.7
TSRNASNP120729235	9	0.0383*	33,5	0.0434*	32.1	0.0652	27.7
TSRNASNP120730012	9	0.0383*	33,5	0.0434*	32.1	0.0652	27.7
TSRNASNP120731088	9	0.0383*	33,5	0.0434*	32.1	0.0652	27.7
TSRNASNP120731107	9	0.0383*	33,5	0.0434*	32.1	0.0652	27.7
TSRNAINDELS120095636	9	0.0383*	33,5	0.0434*	32.1	0.0652	27.7
TSRNASNP120734745	9	0.0383*	33,5	0.0434*	32.1	0.0652	27.7
TSRNAINDELS120095711	9	0.0383*	33,5	0.0434*	32.1	0.0652	27.7
TSRNASNP120735535	9	0.0383*	33.5	0.0434*	32.1	0.0652	27.7
TSRNAINDELS120025818	15	0.1045	36.3	0.4111	16.3	0.0486*	45.4
TSRNASNP120206984	15	0.1045	36.3	0.4111	16.3	0.0486*	45.4
TSRNASNP120273500	17	0.0142*	43.5	0.0053*	52.2	0.0012*	62.8
TSRNASNP120275823	17	0.0142*	43.5	0.0053*	52.2	0.0012*	62.8
TSRNASNP120277213	17	0.0142*	43.5	0.0053*	52.2	0.0012*	62.8
TSRNASNP120278360	17	0.0142*	43.5	0.0053*	52.2	0.0012*	62.8
TSRNASNP120279487	17	0.0142*	43.5	0.0053*	52.2	0.0012*	62.8
TSRNASNP120280865	17	0.0142*	43.5	0.0053*	52.2	0.0012*	62.8
TSRNASNP120275548	17	0.0461*	31.5	0.0379*	33.6	0.0067*	50.2
E.							
		**BV 2010**		**BV 2011**		**BV 2012**	
**Marker**	**Chr**	***p*-value**	**R**^**2**^**(%)**	***p*-value**	**R**^**2**^**(%)**	***p*-value**	**R**^**2**^**(%)**
TSRNAINDELS120073669	6	0.0480*	31.0	0.0244*	38.2	0.08	25.3
TSRNASNP120185697	14	0.2961	9.9	0.0452*	31.7	0.7015	1.4
TSRNASNP120273500	17	0.0017*	60.8	0.0123*	44.8	0.011*	45.9
TSRNASNP120275823	17	0.0017*	60.8	0.0123*	44.8	0.011*	45.9
TSRNASNP120277213	17	0.0017*	60.8	0.0123*	44.8	0.011*	45.9
TSRNASNP120278360	17	0.0017*	60.8	0.0123*	44.8	0.011*	45.9
TSRNASNP120279487	17	0.0017*	60.8	0.0123*	44.8	0.011*	45.9
TSRNASNP120280865	17	0.0017*	60.8	0.0123*	44.8	0.011*	45.9
TSRNASNP120275548	17	0.0274*	37.0	0.0450*	31.7	0.0430*	32.2

Significant associations between SNP and InDel markers and berry quality traits including bunch weight (BWe), berry length (BL), berry width (BWi) and berry volume (BV) were determined (Table 3b, c, d, and e). Markers significantly associated with BWe were found only in chr 17 (Table 3b). Markers significantly associated to BL and BWi were found in chr 6, 8, 15 and 17 (BL) and in chr 6, 9, 15 and 17 for BWi (Table 3c and d). Markers significantly associated with BV were detected in chr 6, 14 and 17 (Table 4e).

**Table 4:** Genotype-phenotype association analysis results including markers that showed significant associations with berry fresh weight (BFW) (A), berry length (BL) (B) and berry width (BWi) (C) determined in a set of 41 grape varieties including table grape varieties (21) as well as core collection varieties (20), harboring a large proportion of the genetic diversity for Prole ‘Orientalis’ of *Vitis vinifera*. *P*-values of associations and variance explained by each SNP/InDel marker (R^2^) are indicated for the GLM models obtained for seasons 2016, 2017 and 2018. Statistically significant associations are in bold and highlighted with an asterisk (*p* < 0.05)

A.							
		**BFW 2016**		**BFW 2017**		**BFW 2018**	
**Marker**	**Chr**	***p*-value**	**R2 (%)**	***p*-value**	**R2 (%)**	***p*-value**	**R2 (%)**
TSRNASNPS120468689	3	0.0123*	22.0	0.0053*	25.1	0.0016*	30.7
TSRNAINDELS120073788	6	0.2951	3.5	0.1426	6.6	0.0333*	13.5
TSRNAINDELS120073728	6	0.0380*	12.9	0.1224	7.3	0.1488	6.5
TSRNASNPS120671218	8	0.0304*	17.9	0.0047*	25.5	0.0126*	21.9
TSRNASNPS120671409	8	0.3371	6.0	0.0499*	15.3	0.0679	14.1
TSRNASNPS120671217	8	0.0639	9.1	0.0388*	11.0	0.0496*	10.2
TSRNASNPS120206839	15	0.0173*	20.5	0.0063*	24.4	0.0678	14.8
B.							
		**BL 2016**		**BL 2017**		**BL 2018**	
**Marker**	**Chr**	***p*-value**	**R2 (%)**	***p*-value**	**R2 (%)**	***p*-value**	**R2 (%)**
TSRNASNPS120468689	3	0.0246*	18.7	0.0137*	21.0	0.0087*	24.8
TSRNASNPS120571906	6	0.6833	0.5	0.0215*	13.4	0.1479	6.0
TSRNAINDELS120073788	6	0.0598	10.9	0.1314	6.7	0.0249*	14.7
TSRNAINDELS120073728	6	0.0474*	12.1	0.4866	1.5	0.1566	6.2
TSRNASNPS120671217	8	0.1058	7.0	0.1517	5.4	0.0384*	11.9
TSRNASNPS120185697	14	0.0813	2.7	0.0250*	18.4	0.2435	8.1
TSRNASNPS120206839	15	0.3131	6.3	0.0310*	17.4	0.1944	9.4
C.							
		**BWi 2016**		**BWi 2017**		**BWi 2018**	
**Marker**	**Chr**	***p*-value**	**R2 (%)**	***p*-value**	**R2 (%)**	***p*-value**	**R2 (%)**
TSRNASNPS120468689	3	0.0264*	18.5	0.0181*	20.3	0.0240*	19.9
TSRNASNPS120671217	8	0.0363*	11.4	0.0863	7.9	0.0543	10.3
TSRNASNPS120671218	8	0.0673	14.1	0.0039*	26.8	0.0160*	21.8
TSRNASNPS120671409	8	0.2070	8.5	0.0410*	16.5	0.0586	15.5
TSRNASNPS120185697	14	0.6119	2.7	0.0479*	15.8	0.1908	9.4
TSRNASNPS120206839	15	0.0077*	23.9	0.0011*	31.8	0.0678	14.8
TSRNASNPS120206217	15	0.0611	14.5	0.0129*	21.8	0.2368	8.2

The previous results are concordant with the findings using Random Forest (RF) analysis **(**Additional file 11: Figure S8) and unbalanced ANOVA (Additional file 12: Table S4), which support complementary information of markers with association to BFW as well as estimating their possible effects on this trait. According to the RF regression results, nine candidate SNPs including seven SNPs located on chr 17, one on chr 14 and one on chr 3, plus one InDel on chr 6 (TSRNAINDELS120073669) were significantly associated to berry weight using 1000 permutations (*p* < 0.05) (Additional file 11: Fig. S8). In fact, six SNPs located in chr 17 (Fig. 2) were the best candidate markers for BFW (*p* < 0.001) (Additional file 11: Fig. S8). The marker contributions to BFW trait estimated by the unbalanced ANOVA suggest that they ranged from 1.4 to 33.0% (Additional file 12: Table S4), showing a non-additive participation.

**Fig. 3:**
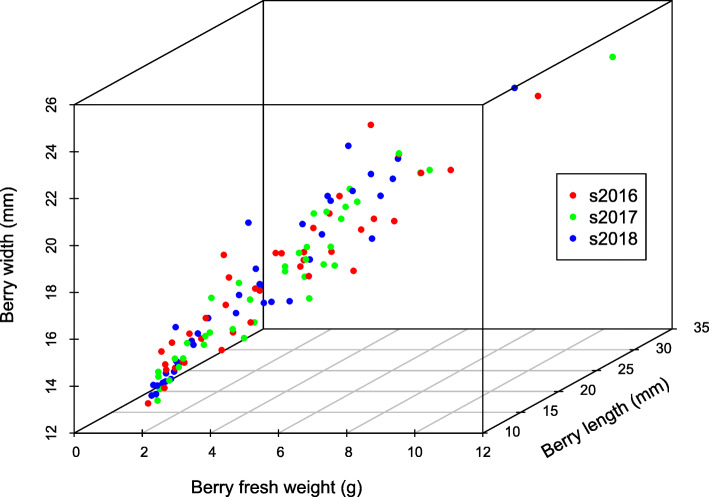
Dispersion plot based on berry fresh weight (BFW), berry length (BL) and berry weight (BWi) traits at harvest, analyzed in a set of 41 grape varieties; including table grape varieties (21), representing the most planted in Chile as well as core collection varieties (20), harboring a large proportion of the genetic diversity for Prole ‘Orientalis’ of *Vitis vinifera.* Phenotypic evaluations were developed during seasons 2016, 2017 and 2018

Subsequently, SNP and InDel markers were evaluated to determine their informativeness in a set of 41 varieties with different genetic backgrounds (Fig. 3), including 21 table grapes varieties representing the most planted in the Chilean vineyard and other countries (Additional file 13: Table S5), and a core collection of 20 varieties harboring a large proportion of the genetic diversity for the Prole ‘Orientalis’ of *V. vinifera* (Additional file 14: Table S6).

Results obtained using a GLM model showed several degrees of association of these markers with BFW (10 to 30%) (Table 4a). In fact, SNPs and InDels significantly associated to BFW (*p* < 0.05) were identified on chr 3, 6, 8 and 15 (Table 4a). Significant associations between SNP and InDel markers and berry length (BL) and berry width (BWi) were also identified (*p* < 0.05) (Table 4b and c), in chr 3, 6, 8, 14 and 15, in the former, and in chr 3, 8, 14 and 15, for the latter.

Association results for BL and BWi traits varied from 11 to 25% and 11 to 32%, corresponding respectively, to the proportion of explained variation. TSRNASNPS120468689 and TSRNASNPS120206839 markers, located in chr 3 and 15, respectively, showed significant associations with the three evaluated traits in at least one season.

### Integration of differentially expressed genes, SNP/InDel polymorphisms and QTLs for berry weight

Physical coordinates were compared between markers and candidate genes to evaluate the co-localization of regions containing candidate SNPs and InDels associated with berry weight and differentially expressed (DE) genes identified in previous transcriptomic analyses [20]. In the eight intervals where the 38 candidate polymorphisms are located, 190 DE genes derived from the comparison between seedless segregants with contrasting berry size were identified. The number of DE genes per interval varied from 15 in chr 15, to 28 in chr 14. These results are summarized in Additional file 15: Table S7.

**Fig. 4:**
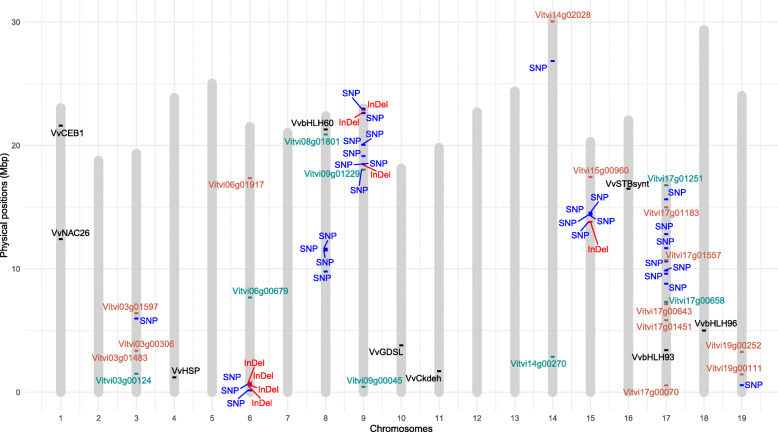
Co-localization of SNP/InDel candidates for berry size and DE genes between large and small berry segregants in *V. vinifera* chromosomes. Thirty-eight candidate polymorphisms for berry weight were identified using a search of structural variants based on a transcriptome experiment [20]. SNPs and InDels are represented in blue and red, respectively. A subset of candidate genes associated with berry weight was also co-localized. Significantly correlated DE genes with PCA-1 are represented in the plot. DE genes up-regulated in SB segregants are represented in orange; DE genes up-regulated in LB segregants are in cyan. DE genes are Vitvi03g00306: F-box protein At2g16365; Vitvi03g01483: AMP-activated protein kinase gamma regulatory subunit putative; Vitvi03g00124: pathogenesis-related protein 5; Vitvi03g01597: secretory protein putative; Vitvi06g00679: GCN5-related N-acetyltransferase (GNAT) family protein; Vitvi06g01917: lichenase; Vitvi08g01801: putative mitochondrial 2-oxoglutarate/malate carrier protein; Vitvi09g01229: anthranilate N-benzoyltransferase protein 2; Vitvi09g00045: 18.6 kDa class III heat shock protein; Vitvi14g00270: copper chaperone; Vitvi14g02028: probable galacturonosyltransferase 13; Vitvi15g00960: O-acyltransferase WSD1; Vitvi17g01557: DEAD-box ATP-dependent RNA helicase 30; Vitvi17g00658: C2H2-type zinc finger transcription factor; Vitvi17g00643: LON peptidase N-terminal domain and RING finger protein 1; Vitvi17g01451: germin-like protein subfamily T member 1; Vitvi17g00070: protein RUPTURED POLLEN GRAIN 1; Vitvi17g01183: probable metal-nicotianamine transporter YSL7; Vitvi17g01251: expansin-A15; Vitvi19g00111: epidermis-specific secreted glycoprotein EP1; Vitvi19g00252: 5′-AMP-activated protein kinase gamma subunit

Figure 4 shows the co-localization between polymorphisms and DE genes. DE genes significantly correlated with component 1 of the principal component analysis (PCA-1), which explains the largest phenotypical variance between large (LB) and small berry (SB) segregants in fruit setting and berry 6–8 mm diameter stages, are shown in orange and cyan, indicating over-expression in SB and LB, respectively.

One SNP and 25 DE genes were identified in chr 3, four of them significatively correlated to PCA-1 corresponding to genes coding for F-box protein At2g16365 (Vitvi03g00306) (0.96), a putative secretory protein (Vitvi03g01597) (0.98), a 5′-AMP-activated protein kinase gamma subunit (Vitvi03g01483) (0.97) as well as a pathogenesis-related protein 5 (Vitvi03g00124) (− 1); the first three over-expressed in SB, and the last over-expressed in LB segregants. We also identified three SNPs in chr 6, plus four InDels and 26 DE genes, two of them significantly correlated with PCA-1, coding for a GCN5-related N-acetyltransferase (GNAT) family protein (Vitvi06g00679) and a lichenase (Vitvi06g01917), whith correlations of − 0.96 and 1, suggesting high expression in LB and SB segregants, respectively (Fig. 4).

Four SNPs and 25 DE genes were found in chr 8, one of them significantly correlated with PCA-1 (− 0.96), coding for a putative mitochondrial 2-oxoglutarate/malate carrier (Vitvi08g01801) suggesting over-expression in LB segregants. Eight SNPs plus three InDels and 25 DE genes were found in chr 9, two of them coding for 18.6 kDa class III heat shock protein (HSP) (Vitvi09g00045) and anthranilate N-benzoyltransferase protein 2 (Vitvi09g01229), both negatively correlated with PCA-1 (− 0.95 and − 0.96) and over-expressed in LB segregants. One SNP and 28 DE genes were identified in chr 14, two of them coding for a copper chaperone (Vitvi14g00270) and a probable galacturonosyltransferase 13 (Vitvi14g02028), both significatively correlated with PCA-1 (− 0.97 and 0.96,), over-expressed respectively in LB and SB segregants. Five SNPs and one InDel, as well as 15 DE genes were identified in chr 15, one of them coding for one O-acyltransferase WSD1 (Vitvi15g00960) highly correlated with PCA-1 (1), indicating its over-expression in SB segregants. Seven SNPs and 25 DE genes were found in chr 17, seven of them significantly correlated with PCA-1. Two DE genes coding for an expansin-A15 (Vitvi17g01251) and the C2H2-type zinc finger transcription factor (Vitvi17g00658) were negatively correlated (− 0.99 and − 0.97), suggesting their over-expression in LB segregants. Also, five genes coding for a DEAD-box ATP-dependent RNA helicase 30 (Vitvi17g01557) (0.99), a probable metal-nicotinamine transporter YSL7 (Vitvi17g01183) (0.99), a germin-like protein subfamily T member 1 (Vitvi17g01451) (0.96), a protein ruptured pollen grain 1 (Vitvi17g00070) (0.97), and a LON peptidase N-terminal domain and RING finger protein 1 (Vitvi17g00643) (0.96) were positively correlated with PCA-1, and over-expressed in SB segregants. Finally, in chr 19 one SNP and 21 DE genes were found, two of them significatively correlated with PCA-1, coding for 5′-AMP-activated protein kinase gamma subunit (Vitvi19g00252) (0.97) and epidermis-specific secreted glycoprotein EP1 (Vitvi19g00111) (0.95), over-expressed in SB segregants (Fig. 4).

## Discussion

### Identification of SNPs and InDels comparing segregants with contrasting phenotypes for berry size

Single Nucleotide Polymorphisms (SNPs) and insertion/deletions (InDels) are the most abundant polymorphisms in every species studied, including plant genomes [55, 56]; both are ubiquitous in genomes, and although less frequent than SNPs, InDels exhibit greater diversity in size [57–59]. The massive utilization of SNP markers in genetic studies in plants is based on their stability, availability, discriminative power and reproducibility [18, 60–63]. InDel markers suitability has been proved in diagnostics and phylogenetic studies in plants. The discovery of novel InDel markers for genomics-assisted breeding application has been recently described based on genome-wide detection in soybean [55], grape [28], peaches [63], chickpea [64], *Brassica rapa* [65], and sweet cherry [66], among others. In grapes, as in other plant species, the most used SNP applications have been focused in genetic diversity evaluation, cultivar identification, linkage maps construction, and recently, phenotype-genotype association studies [18, 27, 30, 60–62].

Our approach was based on the comparison of the transcriptomes of contrasting individuals (segregants of RxS cross) in early stages of berry development (Additional file 1: Table S1). These stages correspond to the first part of the double sigmoid growing curve characteristic for grape berries. Both stages are coincident with vacuolar changes associated to water and organic acids accumulation, resulting in cell expansion and concomitantly determining the final berry size ([18, 26, 67, 68]; revised by 20).

The first approach in this study was directed to the study of SNPs located in DE genes inferred from transcriptomic analyses that compared seedless segregants with contrasting berry sizes [20]. However, these SNPs showed a rather modest association with berry size. Due to this, in a second approach all polymorphisms initially identified were included in the analysis regardless of their genomic location, following the pipeline for filtering and selection presented in Additional file 2: Figure S1. The observed distribution of SNPs between *V. vinifera* chromosomes was similar to that previous reported by [30], the largest number in chr 18 (57,443) and the lowest in chr 17 (26,455) (Additional file 3: Figure S2A).

Considering the results of the analysis of the transcriptomic data, it is relevant to discuss the identification of SNPs (14.3%) and InDels (11.6%) in non-coding regions such as intergenic sequences, some of which could be regulatory regions. The most plausible explanation for this finding could derive from the fact that the grapevine annotation, as in other plant genomes, has been basically an automated process, and because of that possibly harboring some errors. The detection of polymorphisms in intergenic regions is concordant with findings previously reported using similar RNA-seq approaches [43, 69, 70]. These results might imply that some coding regions or even complete genes have not been considered in the current annotations [69]. In fact, several efforts have been developed to curate the *Vitis vinifera* gene model annotation [71], and RNA-Seq data represent a valuable tool that could be useful to confirm gene model. Furthermore, evidence obtained studying the Sultanina genome [28], allowed the identification of many transcripts that did not match to the ones deduced from the reference genome, PN-40024. This inconsistency in part could be explained by the presence of larger than expected transcripts in ‘Sultanina’. Therefore, in using the reference genome for the description of our transcripts, it is possible that some of these “intergenic” sequences are indeed part of genic or regulatory sequences, being in fact “false intergenic” sequences. In any case, considering the difficulties inherent to the annotation of gene functions or determine the functional impact of polymorphisms located at non-coding or regulatory regions, SNPs located in coding regions were selected for further experimental evaluations [43].

Interestingly, SNPs and InDels were also found within intronic regions accordingly with our RNA-Seq data. Even though transcriptomic protocols specifically enhance mature mRNA by the selection of polyadenylated RNA or reduce the presence of ribosomal RNAs [72], the presence of polymorphisms at introns could be explained by pre-mRNAs retention [70, 72], at least in part. Moreover, our results are in agreement with previously reported studies in *Populus nigra* were 15% of SNPs were located at intronic regions, using a similar approach to analyze transcriptomic data for polymorphisms discovery [70]. In fact, one of the InDels described here, located at an intronic region (TSRNAINDELS120073761), was experimentally confirmed (Table 2).

By using a series of filtering approaches, we identify 38 polimorphisms for berry size, 30 SNPs and eight InDels (Tables 1 and 2). They were biallelic and with a MAF of at least 20%. Twenty-one percent of the SNPs were located in exons, with non-synonymous and intragenic changes representing 7.2 and 8.6%, respectively. These results agreed with data reported in maize using HapMap2, in which 21% of the SNPs were present in intragenic regions. This evidence suggests that polymorphisms in coding sequences are less frequent than in non-coding regions [73]. The polymorphisms were located in eight chromosomes of *V. vinifera,* chr 3, 6, 8, 9, 14, 15, 17 and 19 (Fig. 2). Interestingly, 82% of these 38 polymorphisms are in only four chromosomes (6, 9, 15 and 17). In addition, co-localizations of SNPs and InDels in chr 6, 9 and 15 were verified (Fig. 2).

SNPs located in coding regions with non-synonymous and intragenic effects were preferently selected. Functional non-synonymous SNPs were selected in coding regions based on possible effects of these changes over genes function, which may result in changes in aminoacidic residues of the encoded protein, interfering with transcription or protein synthesis [73, 74], as well as changes in structural properties or altered functions in proteins. Due to their significant effect, non-synonymous coding SNPs are considered the most significant drivers of expression regulation, and therefore ideal markers for association studies of quantitative complex traits [73, 75]. However, they exhibit a lower frequency than silent substitutions [73, 74]. Using a similar strategy in rice ca. 200 genes containing non-synonymous SNPs related to *Rhizoctonia solani* were identified [73], and non-synonymous SNPs associated to drought tolerance in maize were also reported [76]. The selection criteria for InDels was less strict due to the much lower number of this type of polymorphism.

### Evaluation of genotype-phenotype association of SNPs/InDels markers and berry size

The platform we selected for the validation SNP and InDel markers was PCR coupled with High Resolution Melting (qPCR-HRM) analysis, a post-PCR detection methodology. It was chosen because of its high sensitivity, speed, accuracy and feasibility for the analysis of double-stranded PCR products dissociation, such as SNPs, InDels [28, 66, 77, 78] and SSRs [79]. The amplification conditions were suited for each SNP/InDel marker (Additional file 8: Table S2, and Additional file 9: Table S3), obtaining reliable HRM and melting curves for segregants and varieties analysed.

Genotype-phenotype association was performed using a subset of 13 segregants of RxS cross, including six segregants with contrasting phenotypes for berry weight which were sequenced, and seven additional segregants with consistently large vs. small berries. Fourteen SNPs distributed in chr 6, 8, 15 and 17 presented significant association with BFW (Table 3a). We observed complementary information from GLM [54], RF and unbalanced ANOVA which confirmed the 10 most important polymorphisms associated to berry weight and estimated their possible effects on this trait. Six of the seven candidate SNPs identified in chr 17, within a region that encompasses 6.8 Mbp, presented a genotype-phenotype association up to 50%; one InDel described in chr 6 (TSRNAINDELS120073669) was significatively relevant for berry weight. The four SNPs identified in chr 8 covering a region of approximately 1.8 Mbp, they presented a genotype-phenotype association up to 50%, but less stable than other markers.

Estimates of the polymorphism contribution in berry weight using unbalanced ANOVA suggested that they ranged between 1.4 to 33% (Additional file 12: Table S4), along with the possible polymorphic linkage associated to specific regions in *V. vinifera* chromosomes. These results could imply that their contribution in berry weight is not additive, something that could be explained considering that ANOVA is an univariant analysis, evaluating one single marker per test, and that possible allelic interactions between markers, such as epistasis, were not considered in the model. Altogether, these results suggest that there is a good chance for both groups of markers, SNPs and InDels, to be used as a substrate to develop selection markers applicable in table grape breeding programs. SNP and InDel markers significantly associated to other quality traits were also identified, including BWe, BL, BWi and BV (Table 3b, c, d, and e), which might suggest a possible interplay between genetic determinants of grape berry-related traits [30].

The multiple evidence observed considering distribution and association of polymorphisms to berry size suggested the presence of regions probably linked to this trait, which explained different levels of the phenotypic variance for berry weight, based on the similar associations observed between polymorphisms, behaving as blocks or QTLs, probably derived from low recombination events.

In relation to the informativeness of SNP and InDel markers on berry weight trait in grapes varieties, our results emphasize the importance of a wide validation using bi-parental populations as well as varieties with different genetic backgrounds. Analysis performed in bi-parental populations are frequently population-specific and need to be tested in wider genetic backgrounds [80].

Significant associations between markers identified on chr 3, 6, 8 and 15 and BFW were determined using a GLM model (10 to 30%) (*p*-value< 0.05) (Table 4a). In addition, our results also showed SNP and InDel markers significantly associated to grape berry-related traits such as BL and BWi (Table 4b and c). The next step will be focused in the evaluation of this set of markers in a broad group of table grapes varieties, in order to select the best combination of them as selection tools for BFW.

### Integration of transcriptome analysis, regions containing candidates SNPs/InDels and QTLs described for berry size

SNP and InDel markers identified in this study were allocated in eight chromosomes of *V. vinifera* (Fig. 2) and associated which genes involved in complex metabolic pathways. These results are concordant with over-represented GO categories identified in these set of genes such as biosynthetic and developmental processes, response to stimulus, transport and localization as well as chromatine remodeling (Additional file 7: Figure S6, and Additional file 10: Figure S7).

The co-localization of regions containing SNP and/or InDel markers with previously reported QTLs for berry weight were analyzed. Significant QTLs have been reported in nine linkage groups. For example, QTLs for berry weight located in LG8, LG11 and LG17 were reported [18], explaining 7 to 31% of phenotypic variance. Our study identified possible co-localizations in three LGs: LG8 and LG17 [18] and LG15 [23].

The physical distance between the QTL described in LG8 by [18], identified in the SxG population and the region where four candidate SNPs for berry weight were located, corresponding to approximately 0.8 Mbp. Five SNPs and one InDel were identified for the QTL associated with berry weight in LG15 [23]. However, the physical coordinates of this QTL were not available in order to estimate the distance between them.

The main QTL located in LG17 was identified in two populations. The first was in seedless table grapes (MTP3140), which included cv. ‘Sultanina’ in one parental’s pedigree, while the second corresponded to the cross of the wine varieties ‘Syrah’ × ‘Grenache’ (SxG). The estimated distance between the QTL interval in LG17 and the region of 6.8 Mbp where the group of six SNPs with highest genotype-phenotype association was 1.9 to 2.1 Mbp, depending on the population analyzed [18]. The identification of this QTL in wine (100% seeded) and table grapes (partially seedless) populations might suggest that the association with berry weight is independent of differential major characteristics such as the presence/absence of seeds. The identification of these QTLs in the same genomic regions for wine and table grape genetic backgrounds strongly suggests that this is a key genetic factor related to berry size.

Thus, the multiple evidence presented in this study confirm previously reported QTLs associated with berry size in LG15 [23], LG8, LG11 and LG17 [18], reinforcing the idea that several genes distributed in different genomic regions (LG) are probably supporting the final expression of this trait.

However, neither SNP nor InDel markers associated with BFW were identified in this study for the QTLs described in LG1 and LG12 [21], LG5 and LG13 [22], and LG18 [24, 34], although the last is related to a major QTL controlling seed content [34]. A plausible explanation could be based in the selection of seedless segregants with contrasting phenotypes for BFW, which might mask the effect of QTL located at LG18.

Significant associations between SNPs and BFW were recently reported in chr 1 related to candidate gene NAC26 [27] and in chr 17, 18 and 19 [30]. Considering the polygenic control of BFW, it has been proposed that different causal polymorphisms might segregate among populations [18, 30].

The observed distribution of SNPs and InDels identified in this study allowed us to find four new regions associated with berry weight in chr 3, 6, 9 and 14. The 38 SNP and InDel markers associated with berry size were distributed in 32 genes; none of which was reported as differentially expressed (DE) between segregants with contrasting phenotypes for berry size in the previous work of our research group [20]. The co-localization of DE genes derived from transcriptome analysis and regions where polymorphisms were identificated allowed us to find at least 190 DE genes to be considered as potentially related to berry size (Fig. 4). Twenty-one of them are particularly interesting due to their relationship with PCA-1 and showing a higher discrimination value between large and small seedless segregants. This group of genes included functional categories associated to transcription regulation, cell wall modification, transport of metal ions, water and organic acids, response to biotic/abiotic stress, protein degradation and protein-kinase activation [20]. Therefore, the co-localization among DE genes and the regions containing SNPs and InDels might support the observed association between candidate polymorphisms and berry size, specially in the case of the 68 DE genes involved in berry size determination [20].

For instance, the single SNP reported at chr 3 was associated to a predicted Ca2 + −dependent phospholipid-binding protein (Table 1) possibly related to annexins which have a relevant role in signalling and adaptation as key factors in cold, oxidative, saline, and abscisic acid (ABA) stress responses [81]. Four DE genes significantly correlated with PCA-1 were also identified in chr 3, coding for a F-box At2g16365 protein, a putative secretory protein, an AMP-activated protein kinase gamma regulatory subunit and a pathogenesis-related protein 5 (Additional file 15: Table S7); the first and third are possibly involved in protein degradation/proteasome and protein modification/kinase, respectively, and the last in stress/defense responses [20].

Chr 6 presented four InDels and three SNPs, co-localizing a gene coding for GCN5-related N-acetyltransferase (GNAT) considered as a possible transcription activator associated with chromatin assembly and DNA replication [82] as well as expansin-A8 possibly associated with cell expansion (Fig. 4). Four SNPs were identified in chr 8, co-localizing putative mitochondrial oxoglutarate/malate transporter protein 2, also up-regulated in large berry segregants, possibly associated with vacuole transport and cell turgor in plants [83] (Fig. 4; Additional file 15: Table S7).

Of the eight SNPs and three InDels identified in chr 9, two genes were up-regulated in large berry segregants coding for heat shock protein 18.6 kDa class III and anthranilate N-benzoyltransferase 2 protein, both related to stress and defense response, co-localized with the polymorphism. Genes coding for WAX2 protein and a putative boron transporter 2, respectively associated to drought in plants [84] and cell wall integrity [85, 86] are also located in the same genomic region (Fig. 4).

Five SNPs and one InDel found in chr 15 where related to genes coding for a beta-fructofuranosidase (invertase), a phosphatidate cytidylyltransferase, a scarecrow-like protein 6, a transketolase 10 and a serine/threonine-protein kinase AtPK2/AtPK19, possibly involved in signaling, activation and biosynthetic process. Seven out of 21 DE genes found in chr 17 were related to berry size determination [20] including expansin-A15, up-regulated in large berry segregants which could be finally responsible for the large berry phenotype and also between SNP variants described in this region and berry size (Fig. 4; Additional file 15: Table S7). SNPs markers and the QTL on LG17 co-localize with a candidate domestication locus of 5 Mbp described by [10] in the study of the genetic structure of *V. vinifera*, supporting the idea of a positive selection of allele(s) for larger fruit, such as the SNPs described here, during the domestication process. The putative orthologue in *V. vinifera* of the gene coding for cytochrome P450 78A, a gene that partially controls fruit weight in tomato and has been associated with its domestication [87], also co-localized in LG17 with the major QTL for berry weight described by [18]. This may suggest conservation of the mechanism of fruit weight control among physiologically and phylogenetically distant species.

Our results are in line with the hypothesis that variations in coding regions as well as in the promotor of the genes are equally important in the determination of complex traits [73]. Equivalent results were observed in maize, where the analysis of quantitative traits showed that 79% of the variations might be explained by SNPs and InDels located in the genes or up to 5 Kb upstream of them [76].

Finally, using an integrated analysis based on RNA-Seq, SNP/InDel search and validation on table grape segregants and grape varieties with different genetic backgrounds, we identified a set of informative and transferable SNP and InDel markers associated with berry size. These results are of interest to achieve better understanding of a complex trait like berry size and could facilitate successful transferability of molecular markers suitable to be integrated in marker-assisted grape breeding programs.

## Conclusions

We identified a set of informative and transferable SNP and InDel markers associated with berry size, using a combined analysis based on RNA-Seq, SNP/InDel search and validation on table grape segregants and grape varieties with different genetic backgrounds. Our results suggest the suitability of SNPs and InDels as candidate markers for berry weight in seedless table grapes. The identification of regions possibly associated with berry weight in chromosomes 8, 15 and 17 was achieved with supporting evidence derived from a transcriptome experiment focused on a SNP/InDel search, as well as a QTL-linkage mapping approach. In addition, new regions possibly associated with berry weight were identified in chromosomes 3, 6, 9 and 14.

## Methods

### Plant material

The population derived of ‘Ruby seedless’ x ‘Sultanina’ cross (RxS, *n* = 139) is planted at the La Platina Experimental Station of the Instituto de Investigaciones Agropecuarias (INIA-La Platina), located in Santiago, Chile; vines are conducted through a trellis system (‘Spanish parron’), grafted over cv. ‘Sultanina’, with two to four replicates being managed under standard conditions as previously described by [20]. This germplasm belongs to the table grape breeding program of INIA. It is available for experimental purposes and, as such, has been used in a number of previous studies [15, 20, 24, 34]. In this case, it has been phenotyped during the 2010 to 2012 seasons for berry size and related subtraits, as well as for a number of traits such as sugar content and titratable acidity, seed weight, rachis architecture and GA-responsiveness, among others.

To evaluate the informativeness of the SNP and InDel markers, a set of 41 varieties with different genetic backgrounds was genotyped, including 21 table grape varieties which include the most planted in Chilean vineyards and other countries (Additional file 13: Table S5) and 20 varieties harboring a large proportion of the genetic diversity for Prole ‘Orientalis’ of *V. vinifera* (Additional file 14: Table S6). This germplasm is established at INIA-La Platina, and is managed by the Genetic Resources Group; it has been phenotyped during several seasons (for instance, from 2016 to 2018) for a number of traits such as berry and seed fresh weight, berry length and berry width, among others (Fig. 3). Vines are conducted through trellis system (‘double cordon’), with three to five replicates per genotype. Grapevine varieties are managed under standard conditions for watering, fertilization, pest and disease control and pruning. All field experiments followed institutional guidelines as well as local legislation.

### Experimental design and sample collection

Both parents plus six segregants of the RxS cross (*N* = 139) with contrasting phenotypes for berry size and weight (Fisher test, *p* < 0.05), i.e., small berry (SB) and large berry (LB), all seedless, were selected and sampled for transcriptome analysis according to [20] (Additional file 1: Table S1). Berry samples were collected at fruit setting (FST) and berries of 6–8 mm diameter (B68), also corresponding to 30 and 45 days after flowering (phenological stages of E-L 27 and E-L 31 [88, 89]). Each genotype was sampled in two or three replicates (clones), later considered as biological replicates.

### RNA extraction, sequencing and data analysis

Pericarp and mesocarp tissues were homogenized and analyzed together for the RNA-Seq experiments, as previously described by [20]. Total RNA was isolated from 3 to 4 g of frozen tissue using the modified hot borate method [90]. RNA integrity was determined using a BioAnalyzer prior to sequencing, selecting samples with RNA Integrity Number (RIN) values over 7.0. Sample sequencing was performed as described [20]. A total of 14 samples of *V. vinifera* were obtained from a group of six segregants and parents from the RxS cross (Additional file 1: Table S1). This subset was part of an experiment previously described [20]. Segregants sharing the same phenotype (SB or LB) were considered as biological replicates in sequencing experiments. Approximately 10 million single-end reads were obtained per sequenced library, with an average length of 50 bp. Quality trimming (Q20) and alignment to the grapevine reference genome (PN40024 12X.v1) [91] was done as described [20]. Multiple reads with more than 20 hits were discarded. The reference grapevine genome and the gene annotation were downloaded from the GENOSCOPE database [92]. Subsequently, the gene annotation was updated using VCost.v3 annotation [93, 94]. The RNA-Seq data used in this study is available at the NCBI’s Sequence Read Achieve [95] (http://www.ncbi.nlm.nih.gov/sra) with SRA Study accession number SRX366617 [96].

### SNP and InDel calling and annotation

A global search of SNP and InDel polymorphisms was developed using reads aligments and the Tophat mapper, with previous removal of PCR-duplicates using remove_duplicates tool from Picard Tools [97, 98] (Additional file 2: Figure S1). The Structural Variant Calling of Genome Analysis Toolkit (GATK) [46] and the Haplotype caller tool were used to identify both SNPs and InDels; for the latter, re-alignments were performed in the regions analyzed (Additional file 2: Figure S1). Polymorphisms are named as ‘TSRNASNP’ or ‘TSRNAINDEL’ using consecutive numbers. Polymorphisms were subsequently filtered using Variant Call Format (VCF 4.0) tools [47]. The following criteria were used to select putative polymorphisms associated with berry size: i) selection of biallelic polymorphisms; ii) no missing data; iii) a minimun separation of 100 bp (‘thin’); and and iv) a MAF (Minor Allele Frequency) of 0.2, previously evaluated at four levels of 0.1, 0.2, 0.3 and 0.4 (Additional file 2: Figure S1). Also, a fixation index (Fst) of 1 or 0 was also applied among libraries of small berry (SB) and large berry segregants (LB), considering the expected genotypical class for each SNP or InDel. For example, the same genotypic class for one SNP or InDel predicted for the three SB indivuals should be different than the genotypic class in LB individuals.

The functional annotation of selected polymorphisms was performed using the Variant Annotation and Effect prediction tool SnpEff software [48, 99], according to the gene model based on the *Vitis vinifera* genome reference (PN40024 12X.1) (Additional file 2: Figure S1). Intragenic and non-synonymous coding SNPs were selected.

SNPs and InDels were then filtered by coverage considering the 11 libraries representative of seedless segregants with contrasting phenotypes for berry size with the ‘Integrative Genomic Viewer’ (IGV) [100–102] (Additional file 2: Figure S1).

### Gene ontology analysis

A gene ontology (GO) enrichment analysis was performed ShinyGO v0.61 tool [49, 50]. The frequency of query genes was compared with the complete reference genome for *V. vinifera* (PN40024), searching for possible enrichment in biological processes. Enrichment analyses were based on a hypergeometric distribution followed by FDR correction. Significant GO terms (*p* < 0.05) were reported.

### Primer design

Specific primers were designed to validate SNPs and InDels using PRIMER 3 software [103] with the following parameters: amplicon length among 80 to 160 bp, primers length between 18 and 23 bp, and annealing temperature (Tm) range within 58 °C–62 °C. Primer dimer formation as well as primer complementarity were checked in silico using Operon software [104]. Primers were synthetized by Integrated DNA Technologies (IDT) (Coralville, Iowa, US). Additional file 8: Table S2 and Additional file 9: Table S3 summarized the primers used in High-Resolution Melting analysis (qPCR-HRM).

### DNA extraction

Total DNA was extracted from young leaves according with [28]. The purified DNA was treated with DNAase-free RNAase A and measured as described [28]. DNA samples with concentrations higher or equal to 40 ng/uL were used in the analyses.

### Experimental validation of SNPs/InDels associated to berry size using High-Resolution Melting analysis (qPCR-HRM)

The validation of SNPs was developed according to the protocol of [28], with modifications. Reactions were optimized for each pair of primers considering annealing temperature (58 °C, 60 °C and 62 °C) and primer concentration (0.2 μM, 0.4 μM, and 0.6 μM) (Additional file 8: Table S2). For InDels, the conditions reported by [77, 78] were optimized considering annealing temperature (58 °C, 60 °C and 62 °C) and primer concentration (0.3 μM, 0.5 μM and 0.7 μM) (Additional file 9: Table S3). Real time PCR and HRM reactions were done and scored as described by [28]. Reactions were based on EvaGreen®, using a primer concentration that ranged from 0.2 μM to 1.1 μM, plus 1 ng of DNA template in a total reaction volume of 10 μL. Three replicates were used per individual. Genotypes were manually assigned by examining melting and HRM plots previously normalized and derivatized.

Polymorphisms were confirmed by sequencing amplification products (Macrogen Inc., Korea), and sequenced samples were used as controls. The qPCR-HRM amplicons of varieties ‘Sultanina’ and ‘Ruby seedless’ as well as RxS segregants were quantified using the method described by [28]; samples were sequenced only if DNA concentration was higher or equal to 20 ng/uL. SNP/InDel amplified fragments were aligned to the reference grapevine genome using Sequencher software (Gene Codes Corporation).

### Genotype-phenotype association analysis between SNPs/InDels and berry weight using random Forest analysis, GLM model, unbalanced variance analysis (ANOVA)

Three methods were used to determine significant associations between SNP/InDel markers and berry quality traits: GLM based on TASSEL, Random Forest and unbalanced variance analysis **(**ANOVA), using a subset of 13 RxS segregants. Phenotypic data were normalized using logarithmic transformation to fulfill the normality assumption in the statistical analyses [27].

Genotype-phenotype association analysis were performed using SNP/InDel markers for berry fresh weight (BFW), bunch weight (BWe), berry length (BL), berry width (BWi) and berry volume (BV), in seasons 2010, 2011 and 2012, using the Global Linear Model (GLM) implemented in software TASSEL v.5.0 [54].. The group of 13 seedless segregants from RxS cross and both parentals were genotyped with using the 38 SNP and eight InDel markers. The group of segregants included the six segregants previously sequenced as well as seven segregants of the RxS cross, all of them phenotyped during three consecutive seasons. GLM finds the ordinary least squares solution for each marker-trait combination [105, 106]. Variance explained and *p*-values for each SNP/InDel marker were recorded. Subsequently, a *Random Forest* (RF) analysis as well as unbalanced ANOVA were developed in order to determine significant associations between SNP/InDel markers and BFW. RF corresponds to a multivariate analysis mainly used for statistical classification and regression. An RF model is an ensemble of unpruned classification or regression trees created using bootstrap samples of the training data and random feature selection in tree induction [107]. The importance of the variable was estimated for SNPs and InDels markers with association to berry fresh weight, which can be interpreted according to the relative ranking of the significant predictors [108] based on the RF regression of the phenotypic values observed in the group of 13 seedless segregants with contrasting phenotypes for berry weight and the genotypical data. The analysis was performed using 1000 permutations test, the ‘*randomForest*‘package of R statistical software and a *p*-value of 0.05.

Then a one-way variance analysis (ANOVA) was performed using unbalanced data and the group of 38 candidate markers in order to determine their individual effect (R^2^) on berry weight according with the proposed model, using the *stats* package of R (R 3.6.2) [109]. The coefficient of determination of the model was performed using R and considered the sum of mean squares,

$$ yij=\mu +\mathrm{xj}+\varepsilon \mathrm{ij}. $$

where *yij* is the observation *i* of berry fresh weight phenotype according to the genotype of *j* for marker *x*; *μ* is the overall mean; *xj* represents genotype *j* of the marker *x*; and *ε* is the error term.

### Estimation of the effect of the SNP and InDel markers associated with berry weight using GLM model and a set of 41 grapes varieties

Genotype-phenotype association analyses were performed using phenotype and genotype data derived from a set of 41 varieties and SNP/InDel markers for berry fresh weight (BFW), berry length (BL) and berry width (BWi), in seasons 2016, 2017 and 2018, using the Global Linear Model (GLM) implemented in software TASSEL v.5.0 [54]. For each marker-trait combination, GLM finds the ordinary least squares solution as described in [105]. Variance explained and *p*-values for each SNP/InDel marker were recorded.

## Supplementary information

**Additional file 1: Table S1.** Summary of RNA sequencing data used for global identification of SNPs and InDels. Samples included RxS segregants with contrasting phenotypes for berry size, i.e. small berry (SB) and large berry (LB), and both parents, ‘Ruby seedless’ and ‘Sultanina’. Berries were collected at fruit setting and berry of 6–8 mm stages, according to [20].

**Additional file 2: Figure S1.** Bioinformatics workflow for the identification and characterization of SNP and InDel molecular markers derived from RNA-Seq data, associated with berry weight trait in *Vitis vinifera.*

**Additional file 3: Figure S2A, S2B.** Distribution of SNP (A) and InDel (B) polymorphisms along *V. vinifera* chromosomes.

**Additional file 4: Figure S3.** SNP distribution along the *Vitis vinifera* genome according to SnpEffect analysis based on a gene model for *V. vinifera* (PN40024).

**Additional file 5: Figure S4.** InDel distribution along the *Vitis vinifera* genome according to SnpEffect analysis based on a gene model for *V. vinifera* (PN40024).

**Additional file 6: Figure S5.** SNP and InDel density determined in *V. vinifera* chromosomes. Density was estimated considering the total observed polymorphisms per Kb.

**Additional file 7: Figure S6.** Hierarchical tree summarizing over-represented gene ontology (GO) categories identified in a set of 232 genes containing 382 SNP markers. Dots at branches represent significant FDR values.

**Additional file 8: Table S2.** Primers designed for the validation of 30 SNPs and the subsequent genotyping of seedless segregants from RxS population and table grape varieties, based on High Resolution Melting analysis (qPCR-HRM).

**Additional file 9: Table S3.** Primers designed for the validation of eight InDels and the subsequent genotyping of seedless segregants from RxS crossing and table grape varieties, based on High Resolution Melting analysis (qPCR-HRM).

**Additional file 10: Fig. S7.** Hierarchical tree summarizing over-represented gene ontology (GO) categories, identified in a set of 32 genes containing SNP and InDel markers. Dots at branches represent significant FDR values.

**Additional file 11: Figure S8.** Random Forest regression analysis was applied to identify the most important SNP or/and InDel markers over the phenotype variation. Analysis was performed using observed genotypes of 38 candidate polymorphisms and the 13 segregants with known phenotype for berry weight, including the parents ‘Ruby seedless’ and ‘Sultanina’. Berry weight (BW) was considered as the response variable; 1000 permutations were used and decision trees were obtained, selecting a consensus. Red line indicates significant polymorphisms. At least 10 markers representing nine SNPs and one InDel were significantly associated with BW (*p* < 0.05).

**Additional file 12: Table S4.** Unbalanced analysis of variance (ANOVA) to analyze possible effects of 30 SNP and eight InDels candidates associated with berry weight.

**Additional file 13: Table S5.** Collection of 21 table grape varieties, representative of table grape diversity cultivated in Chile.

**Additional file 14: Table S6.** International collection of 20 varieties, representative of genetic diversity of *Vitis vinifera* Prole ‘*Orientalis’.*

**Additional file 15: Table S7.** Co-localization of 38 SNP/InDel markers associated with berry weight, and differentially expressed genes (DE genes) between large and small berry segregants, previously reported by [20]. The total of DE genes located in genomic regions overlapping physical coordinates of SNP/InDel markers is shown, encompassing eight chromosomes. DE genes significantly correlated with PCA-1 component, are highlighted in yellow.

## Data Availability

The RNA-Seq data used in this study is available at the NCBI’s Sequence Read Achieve (http://www.ncbi.nlm.nih.gov/sra) with the SRA Study accession number SRX366617.

## Abbreviations

SNP(s): Single nucleotide polymorphism(s)
SSR: Simple sequence repeat
InDel(s): Insertion(s) and deletion(s)
SB: Small berry segregant
LB: Large berry segregant
chr: Chromosome
MAS: Marker-assisted selection
SV(s): Structural variant(s)
CDS(s): Coding DNA sequence
GO: Gene ontology
qPCR-HRM: High-Resolution Melting analysis
QTL: Quantitative trait loci
LG: Linkage group; bp: base pairs

## References

[1] FAOSTAT, 2019. Food and agriculture Organization of United Nations. Statistics division. Available at: http://faostat3.fao.org. Accessed 15 Apr 2019.

[2] Chen N, Wang LC, Fang LC, Liang SH, Wu BH (2015). Construction of a high-density genetic map and QTLs mapping for sugars and acids in grape berries. BMC Plant Biol.

[3] Yamada M, Sato A (2016). Advances in table grape breeding in Japan. Breed Sci.

[4] Koyama K, Kamigakiuchi H, Iwashita K, Mochioka R, Goto-Yamamoto N (2017). Polyphenolic diversity and characterization in the redepurple berries of east Asian wild Vitis species. Phytochemistry..

[5] Bigard A, Berhe DT, Maoddi E, Sire Y, Boursiquot JM, Ojeda H (2018). *Vitis vinifera* L fruit diversity to breed varieties anticipating climate changes. Front Plant Sci.

[6] Alleweldt G, Dettweiler E (1994). The genetic resources of Vitis: World list of grapevine collections.

[7] Lacombe T, Boursiquot JM, Laucou V, Di Vecchi-Staraz M, Péros JP, This P (2013). Large-scale parentage analysis in an extended set of grapevine cultivars (*Vitis vinifera* L.). Theor Appl Genet.

[8] Aradhya MK, Dangl GS, Prins BH, Boursiquot JM, Walker A, Meredith CP (2003). Genetic structure and differentiation in cultivated grape *Vitis vinifera* L. Genet Res.

[9] Salmaso M, Faes G, Segala C, Stefanini M, Salakhutdinov L, Zyprian E (2004). Genome diversity and gene haplotypes in the grapevine (*Vitis vinifera*), as revealed by single nucleotide polymorphisms. Mol Breed.

[10] Myles S, Boyko AR, Owens CL, Brown PJ, Grassi F, Aradhya MK (2011). Genetic structure and domestication history of the grape. Proc Natl Acad Sci U S A.

[11] Di Gaspero G, Cattonaro F (2010). Application of genomics to grapevine improvement. Aust J Grape Wine R.

[12] Costenaro da Silva D, Passaia G, Henriques JAP, Margis R, Pasquali G (2010). Identification and expression analysis of genes associated with the early berry development in the seedless grapevine (*Vitis vinifera* L.) cultivar Sultanine. Plant Sci.

[13] Gouthu S, Deluc L (2015). Timing of ripening initiation in grape berries and its relationship to seed content and pericarp auxin levels. BMC Plant Biol.

[14] Acheampong AK, Zheng C, Halaly T, Giacomelli L, Takebayashi Y, Jikumaru Y (2017). Abnormal endogenous repression of GA signaling in a seedless table grape cultivar with high berry growth response to GA application. Front Plant Sci.

[15] Correa J, Ravest G, Laborie D, Mamani M, Torres E, Muñoz C, et al. Quantitative trait loci for the response to gibberellic acid of berry size and seed mass in tablegrape (*Vitis vinifera* L.). Aust J Grape Wine R. 2015;21:496–507. 10.1111/ajgw.12141.

[16] Dokoozlian NK, Peacock WL (2001). Gibberellic acid applied at bloom reduces fruit set and improves size of ‘crimson seedless’ table grapes. HortScience..

[17] Giacomelli L, Rota-Stabelli O, Masuero D, Acheampong AK, Moretto M, Caputi L (2013). Gibberellin metabolism in *Vitis vinifera* L. during bloom and fruit-set: functional characterization and evolution of grapevine gibberellin oxidases. J Exp Bot.

[18] Doligez A, Bertrand Y, Farnos M, Grolier M, Romieu C, Esnault F (2013). New stable QTLs fir berry weight do not colocalize with QTLs for seed traits in cultivated grapevine (*Vitis vinifera* L.). BMC Plant Biol.

[19] Houel C, Martin-Magniette ML, Nicolas SD, Lacombe T, Le Cunff L, Franck D (2013). Genetic variability of berry size in the grapevine (*Vitis vinifera* L.). Aust J Grape Wine R.

[20] Muñoz-Espinoza C, Di Genova A, Correa J, Silva R, Maass A, González-Agüero M (2016). Transcriptome profiling of grapevine seedless segregants during berry development reveals candidate genes associated with berry weight. BMC Plant Biol.

[21] Costantini L, Battilana J, Lamaj F, Fanizza G, Grando MS (2008). Berry and phenology-related traits in grapevine (*Vitis vinifera* L.): from quantitative trait loci to underlying genes. BMC Plant Biol.

[22] Fischer BM, Salakhutdinov I, Akkurt M, Eibach R, Edwards KJ, Töpfer R (2004). Quantitative trait locus analysis of fungal disease resistance factors on a molecular map of grapevine. Theor Appl Genet.

[23] Cabezas JA, Cervera MT, Ruiz-Garcia L, Carreño J, Martinez-Zapater JM. A genetic analysis of seed and berry weight in grapevine. Genome. 2006;49:1572–85. 10.1139/g06-122 17426772

[24] Mejía N, Gebauer M, Muñoz L, Hewstone N, Muñoz C, Hinrichsen P (2007). Identification of QTLs for seedlessness, berry size, and ripening date in a seedless x seedless table grape progeny. Am J Enol Vitic.

[25] Jiang GL. Molecular Markers and Marker-Assisted Breeding in Plants. In: Plant Breeding from Laboratories to Fields. Edited by Sven Bode Andersen. IntechOpen, London. 2013. 10.5772/52583.

[26] Nicolas P, Lecourieux D, Gomès E, Delrot S, Lecourieux F (2013). The grape berry-specific basic helix–loop–helix transcription factor VvCEB1 affects cell size. J Exp Bot.

[27] Tello J, Torres-Pérez R, Grimplet J, Carbonell-Bejerano P, Martínez-Zapater JM, Ibáñez J (2015). Polymorphisms and minihaplotypes in the VvNAC26 gene associate with berry size variation in grapevine. BMC Plant Biol.

[28] Di Genova A, Miyasaka Almeida A, Muñoz-Espinoza C, Vizoso P, Travisany D, Moraga C (2014). Whole genome comparison between table and wine grapes reveals a comprehensive catalog of structural variants. BMC Plant Biol.

[29] Matthews MA, Nuzzo V (2007). Berry size and yield paradigms on grapes and wines quality. Acta Hortic.

[30] Guo DL, Zhao HL, Li Q, Zhang GH, Jiang JF, Liu CH (2019). Genome-wide association study of berry- related traits in grape [*Vitis vinifera* L.] based on genotyping-by-sequencing markers. Hortic Res.

[31] Eibach R, Zyprian E, Welter L, Töpfer R (2007). The use of molecular markers for pyramiding resistance genes in grapevine breeding. Vitis..

[32] Herzog E, Töpfer R, Hausmann L, Eibach R, Frisch M (2013). Selection strategies for marker-assisted background selection with chromosome-wise SSR multiplexes in pseudo-backcross programs for grapevine breeding. Vitis..

[33] Emanuelli F, Sordo M, Lorenzi S, Battilana Y, Grando MS (2014). Development of user friendly functional molecular markers for VvDXS gene conferring Muscat flavor in grapevine. Mol Breed.

[34] Mejía N, Soto B, Guerrero M, Casanueva X, Houel C, Miccono MA (2011). Molecular, genetic and transcriptional evidence for a role of VvAGL11 in stenospermocarpic seedlessness in grapevine. BMC Plant Biol.

[35] Emanuelli F, Battilana Y, Constantini L, Le Cunff L, Boursiquot JM, This P, Grando M (2010). A candidate gene association study on muscat flavor in grapevine (*Vitis vinifera* L.). BMC Plant Biol.

[36] Riaz S, Tenscher AC, Ramming DW, Walker MA (2011). Using a limited mapping strategy to identify major QTLs for resistance to grapevine powdery mildew (*Erysiphe necator*) and their use in marker-assisted breeding. Theor Appl Genet.

[37] Barba P, Cadle-Davidson L, Harriman J, Glaubitz JC, Brooks S, Hyma K (2014). Grapevine powdery mildew resistance and susceptibility loci identified on a high-resolution SNP map. Theor Appl Genet.

[38] Amrine KCH, Blanco-Ulate B, Riaz S, Pap D, Jones L, Figueroa-Balderas R (2015). Comparative transcriptomics of central Asian *Vitis vinifera* accessions reveals distinct defense strategies against powdery mildew. Hortic Res.

[39] Morozova O, Marra MA (2008). Applications of next-generation sequencing technologies in functional genomics. Genomics..

[40] Mammadov J, Aggarwal R, Buyyarapu R, Kumpatla S. SNP markers and their impact on plant breeding. Int J Plant Genomics. 2012;728398. 10.1155/2012/728398. 10.1155/2012/728398 PMC3536327 23316221

[41] Wang W, Mauleon R, Hu Z, Chebotarov D, Tai S, Wu Z (2018). Genomic variation in 3,010 diverse accessions of Asian cultivated rice. Nature..

[42] Iquebal MA, Sharma P, Jasrotia RS, Jaiswal A, Kaur A, Saroha M, et al. RNAseq analysis reveals drought- responsive molecular pathways with candidate genes and putative molecular markers in root tissue of wheat. Sci Rep. 2019;9:13917. 10.1038/s41598-019-49915-2. 10.1038/s41598-019-49915-2 PMC6763491 31558740

[43] Adetunji MO, Lamont SJ, Abasht, B., Schmidt, C.J. Variant analysis pipeline for accurate detection of genomic variants from transcriptome sequencing data. PLoS One 2019;14:e0216838. 10.1371/journal.pone.0216838. 10.1371/journal.pone.0216838 PMC6756534 31545812

[44] Thakur O, Randhawa GS (2018). Identification and characterization of SSR, SNP and InDel molecular markers from RNA-Seq data of guar (*Cyamopsis tetragonoloba*, L. Taub.) roots. BMC Genomics.

[45] Structural Variant Calling of Genome Analysis Toolkit (GATK). 2010. Available at: http://www.broadinstitute.org/gatk. Accesed 2 Aug 2015.

[46] Trapnell C, Pachter L, Salzberg SL (2009). TopHat: discovering splice junctions with RNA-Seq. Bioinformatics..

[47] Danecek P, Auton A, Abecasis G, Albers CA, Banks E, DePristo MA, et al and 1000 genomes project analysis group. The variant call format and VCFtools. Bioinformatics. 2011;27:2156–8. 10.1093/bioinformatics/btr330 PMC3137218 21653522

[48] Cingolani P, Platts A, Wang LL, Coon M, Nguyen T, Wang L (2012). A program for annotating and predicting the effects of single nucleotide polymorphisms, SnpEff: SNPs in the genome of *Drosophila melanogaster* strain w1118; iso-2; iso-3. Fly..

[49] ShinyGO v0.61 tool. 2019. Available at: http://ge-lab.org/go. Accesed 30 Oct 2019.

[50] Ge SX, Jung D, Yao R. ShinyGO: a graphical gene-set enrichment tool for animals and plants. Bioinformatics. 2019:btz931. 10.1093/bioinformatics/btz931. 10.1093/bioinformatics/btz931 PMC7178415 31882993

[51] Wiewiórka M, Szmurło A, Kúsmirek W, Gambin T (2019). SeQuiLa-cov: a fast and scalable library for depth of coverage calculations. Gigascience..

[52] Pruzinská A, Tanner G, Anders I, Roca M, Hörtensteiner S (2003). Chlorophyll breakdown: pheophorbide a oxygenase is a Rieske-type iron-sulfur protein, encoded by the accelerated cell death 1 gene. Proc Natl Acad Sci U S A.

[53] Tanaka M, Kikuchi A, Kamada H (2008). The Arabidopsis histone deacetylases HDA6 and HDA19 contribute to the repression of embryonic properties after germination. Plant Physiol.

[54] TASSEL v.5.0. 2007. Available at: https://www.maizegenetics.net/tassel. Accesed 21 May 2017.

[55] Song X, Wei H, Cheng W, Yang S, Zhao Y, Li X (2015). Development of InDel Markers for genetic mapping based on whole genome resequencing in soybean. G3 (Bethesda).

[56] Pena HB, Pena SD (2012). Automated genotyping of a highly informative panel of 40 short insertion-deletion polymorphisms resolved in polyacrylamide gels for forensic identification and kinship analysis. Transfus Med Hemother.

[57] Mullaney JM, Mills RE, Pittard WS, Devine SE (2010). Small insertions and deletions (InDels) in human genomes. Hum Mol Genet.

[58] Pacurar DI, Pacurar ML, Street N, Bussell JD, Pop TI, Gutierrez L (2012). A collection of INDEL markers for map-based cloning in seven Arabidopsis accessions. J Exp Bot.

[59] Montgomery SB, Goode DL, Kvikstad E, Albers CA, Zhang ZD, Mu XJ (2013). The origin, evolution, and functional impact of short insertion–deletion variants identified in 179 human genomes. Genome Res.

[60] Lijavetzky D, Cabezas JA, Ibañez A, Rodríguez V, Martínez-Zapater JM (2007). High throughput SNP discovery and genotyping in grapevine (*Vitis vinifera* L.) by combining a re-sequencing approach and SNPlex technology. BMC Genomics.

[61] Cabezas JA, Ibáñez J, Lijavetzky D, Vélez D, Bravo G, Rodríguez V (2011). A 48 SNP set for grapevine cultivar identification. BMC Plant Biol.

[62] Emanuelli F, Lorenzi S, Grzeskowiak L, Catalano V, Stefanini M, Troggio M (2013). Genetic diversity and population structure assessed by SSR and SNP markers in a large germplasm collection of grapes. BMC Plant Biol.

[63] Lambert P, Campoy JA, Pacheco I, Mauroux JB, Da Silva Linge C, Micheletti D, et al. Identifying SNP markers tightly associated with six major genes in peach [*Prunus persica* (L.) Batsch] using a high-density SNP array with an objective of marker-assisted selection (MAS). Tree Genet Genomes. 2016;12:121. 10.1007/s11295-016-1080-1.

[64] Das S, Upadhyaya HD, Bajaj D, Kujur A, Badoni S, Kumar LV, et al. Deploying QTL-seq for rapid delineation of a potential candidate gene underlying major trait-associated QTL in chickpea. DNA Res 2015;22:193–203. 10.1093/dnares/dsv004. 10.1093/dnares/dsv004 PMC4463844 25922536

[65] Liu L, Qu C, Wittkop B, Yi B, Xiao Y, He Y (2013). A high-density SNP map for accurate mapping of seed fibre QTL in *Brassica napus* L. PLoS One.

[66] Muñoz-Espinoza C, Espinosa E, Bascuñán R, Tapia S, Meneses C, Miyasaka-Almeida A (2017). 2017. Development of a molecular marker for self-compatible S4’ haplotype in sweet cherry (*Prunus avium* L.) using high resolution melting. Plant Breed.

[67] Coombe BG (1992). Research on development and ripening of the grape berry. Am J Enol Vitic.

[68] Coombe BG, McCarthy MG (2000). Dynamics of grape berry growth and physiology of ripening. Aust J Grape Wine R.

[69] Kim JE, Oh SK, Lee JH, Lee BM, Jo SH. Genome-wide SNP calling using next generation sequencing data in tomato. Mol Cell 2014;37:36–42. 10.14348/molcells.2014.2241. 10.14348/molcells.2014.2241 PMC3907006 24552708

[70] Rogier O, Chateigner A, Amanzougarene S, Lesage-Descauses MC, Balzergue S, Brunaud V, et al. Accuracy of RNAseq based SNP discovery and genotyping in *Populus nigra*. BMC Genomics 2018;19:909. 10.1186/s12864-018-5239-z. 10.1186/s12864-018-5239-z PMC6291945 30541448

[71] Grimplet J, Van Hemert J, Carbonell-Bejerano P, Díaz-Riquelme J, Dickerson J, Fennell A, et al. Comparative analysis of grapevine whole-genome gene predictions, functional annotation, categorization and integration of the predicted gene sequences. BMC Res Notes 2012;5. 10.1186/1756-0500-5-213. 10.1186/1756-0500-5-213 PMC3419625 22554261

[72] Gaidatzis D, Burger L, Florescu M, Stadler MB. Analysis of intronic and exonic reads in RNA-seq data characterizes transcriptional and post-transcriptional regulation. Nat Biotechnol. 2015;33:722–9. 10.1038/nbt.3269. 10.1038/nbt.3269 26098447

[73] Xu J, Yuan Y, Xu Y, Zhang G, Guo X, Wu F (2014). Identification of candidate genes for drought tolerance by whole-genome resequencing in maize. BMC Plant Biol.

[74] Cargill M, Altshuler D, Ireland J, Sklar P, Ardlie K, Patil N, et al. Characterization of single-nucleotide polymorphisms in coding regions of human genes. Nat Genet 1999;22:231–8. 10.1038/10290. 10.1038/10290 10391209

[75] Xiao Y, Liu H, Wu L, Warburton M, Yan J. Genome-wide association studies in maize: praise and stargaze. Mol Plant 2017;10:359–74. 10.1016/j.molp.2016.12.008. 10.1016/j.molp.2016.12.008 28039028

[76] Xue Y, Warburton ML, Sawkins M, Zhang X, Setter T, Xu Y (2013). Genome-wide association analysis for nine agronomic traits in maize under well-watered and water-stressed conditions. Theor Appl Genet.

[77] Rouleau E, Lefol C, Bourdon V, Coulet F, Noguchi T, Soubrier F (2009). Quantitative PCR high-resolution melting (qPCR- HRM) curve analysis, a new approach to simultaneously screen point mutations and large rearrangements: application to MLH1 germline mutations in lynch syndrome. Hum Mutat.

[78] Borun P, Bartkowiak A, Banasiewicz T, Nedoskytko B, Nowakowska D, Teisseyre M (2013). High resolution melting analysis as a rapid and efficient method of screening for small mutations in the STK11 gene in patients with Peutz-Jeghers syndrome. BMC Med Genet.

[79] Distefano G, Caruso M, La Malfa S, Gentile A, Wu SB (2012). High resolution melting analysis is a more sensitive and effective alternative to gel-based platforms in analysis of SSR-an example in citrus. PLoS One.

[80] Tello J, Torres-Perez R, Grimplet J, Ibañez J (2016). Association analysis of grapevine bunch traits using a comprehensive approach. Theor Appl Genet.

[81] Mortimer JC, Laohavisit A, Macpherson N, Webb A, Brownlee C, Battey NH, et al. Annexins: multifunctional components of growth and adaptation. J Exp Bot. 2008;59:533–44. 10.1093/jxb/erm344. 10.1093/jxb/erm344 18267940

[82] Dyda F, Klein DC, Hickman AB (2000). GCN5-related N-acetyltransferases: a structural overview. Annu Rev Biophys Biomol Struct.

[83] Martínez-Esteso MJ, Sellés-Marchart S, Lijavetzky D, Pedreño MA, Bru-Martinez R (2011). A DIGE-based quantitative proteomic analysis of grape berry flesh development and ripening reveals key events in sugar and organic acid metabolism. J Exp Bot.

[84] Chen X, Goodwin SM, Boroff VL, Liu X, Jenks MA (2003). Cloning and characterization of the WAX2 gene of Arabidopsis involved in cuticle membrane and WAX production. Plant Cell.

[85] Filiz E (2013). In silico characterization of boron transporter (BOR1) protein sequences in Poaceae species. J BioSci Biotech.

[86] Fitzpatrick KL, Reid RJ (2010). The ever expanding role of aquaglyceroporins. Confirmation of protein-facilitated boron transport. Plant Signal Behav.

[87] Chakrabarti M, Zhang N, Sauvage C, Muños S, Blanca J, Cañizares J (2013). A cytochrome P450 regulates a domestication trait in cultivated tomato. Proc Natl Acad Sci U S A.

[88] Coombe BG (1995). Adoption of a system for identifying grapevine growth stages. Aust J Grape Wine R.

[89] Lorenz DH, Eichhorn KW, Bleiholder H, Klose R, Meier U, Weber E (1994). Phaenologische Entwicklungsstadien der Weinrebe (*Vitis vinifera* L. ssp. vinifera). Codierung und Beschreibungnach der erweiterten BBCH-Skala. Viticultural Enological Sci.

[90] Gudenschwager O, González-Agüero M, Defilippi BG (2012). A general method for high-quality RNA isolation from metabolite-rich fruits. S Afr J Bot.

[91] Jaillon O, Aury JM, Noel B, Policriti A, Clepet C, Casagrande A (2007). The grapevine genome sequence suggests ancestral hexaploidization in major angiosperm phyla. Nature..

[92] GENOSCOPE database. Available at: http://www.genoscope.cns.fr/externe/GenomeBrowser/Vitis/.

[93] GENOSCOPE database. 2007. Available at: http://www.genoscope.cns.fr/externe/GenomeBrowser/Vitis/. Accesed 8 Mar 2015.

[94] Canaguier A, Grimplet J, Di Gaspero G, Scalabrind S, Duchêne E, Choisne N (2017). A new version of the grapevine reference genome assembly (12X.v2) and of its annotation (VCost.v3). Genom Data.

[95] NCBI’s Sequence Read Archive. 2009. Available at: http://www.ncbi.nlm.nih.gov/sra. Accesed 15 Apr 2015.

[96] González-Agüero M, García-Rojas M, Di Genova A, Correa J, Maass A, Orellana A, et al. Identification of two putative reference genes from grapevine suitable for gene expression analysis in berry and related tissues derived from RNA-Seq data. BMC Genomics. 2013;14:878. 10.1186/1471-2164-14-878 PMC3878734 24330674

[97] Li H, Handsaker B, Wysoker A, Fennell T, Ruan J, Homer N (2009). 1000 genome project data processing subgroup. The sequence alignment/map format and SAMtools. Bioinformatics..

[98] Picard Toolkit. Broad Institute, GitHub. Repository. 2019. Available at: http://broadinstitute.github.io/picard/. Accesed 17 Sept 2015.

[99] Variant Annotation and Effect prediction tool SnpEff software. 2012. Available at: http://snpeff.sourceforge.net/. Accesed 1 Dec 2015.

[100] Integrative Genomic Viewer (IGV). 2011. Available at: http://www.broadinstitute.org/software/igv. Accesed 5 Feb 2016.

[101] Robinson JT, Thorvaldsdóttir H, Winckler W, Guttman M, Lander ES, Getz G (2011). Nat Biotechnol.

[102] Thorvaldsdóttir H, Robinson JT, Mesirov JP (2012). Integrative genomics viewer (IGV): high-performance genomics data visualization and exploration. Brief Bioinform.

[103] PRIMER 3. Available at: (http://frodo.wi.mit.edu/).

[104] Untergasser A, Cutcutache I, Koressaar T, Ye J, Faircloth BC, Remm M, et al. Primer3 - new capabilities and interfaces. Nucleic Acids Res. 2012;40(15):e115. 10.1093/nar/gks596 PMC3424584 22730293

[105] Oligo Analysis Tool. Operon software. 2017. Available at: http://www.operon.com/tools/oligo-analysis-tool.aspx. Accesed 15 Dec 2017.

[106] Bradbury PJ, Zhang Z, Kroon DE, Casstevens TM, Ramdoss Y, Buckler ES (2007). TASSEL: software for association mapping of complex traits in diverse samples. Bioinformatics..

[107] Acharjee A, Kloosterman B, de Vos RC, Werij JS, Bachem CW, Visser RG (2011). Data integration and network reconstruction with ~omics data using random Forest regression in potato. Anal Chim Acta.

[108] Strobl C, Malley J, Tutz G (2009). An introduction to recursive partitioning: rational, application, and characteristics of classification and regression trees, bagging, and random forests. Psychol Methods.

[109] R Core Team (2012). R: a language and environment for statistical computing.

