# Supplementary material for: Identification of SNPs and InDels associated with berry size in table grapes integrating genetic and transcriptomic approaches
Source: BMC Plant Biol. 2020 Aug 3;20:365. doi: 10.1186/s12870-020-02564-4 (PMC7397606; doi:10.1186/s12870-020-02564-4)
Supplement: Supplementary file 1 — Additional file 1: Table S1. Summary of RNA sequencing data used for global identification of SNPs and InDels. Samples included RxS segregants with contrasting phenotypes for berry size, i.e. small berry (SB) and large berry (LB), and both parents, ‘Ruby seedless’ and ‘Sultanina’. Berries were collected at fruit setting and berry of 6–8 mm stages, according to [20]. [file 12870_2020_2564_MOESM1_ESM.docx]

**Supplementary Table S1.** Summary of RNA sequencing data used for global identification of SNPs and InDels. Samples included RxS segregants with contrasting phenotypes for berry size, *i.e*. small berry (SB) and large berry (LB), and both parents, 'Ruby seedless' and 'Sultanina'. Berries were collected at fruit setting and berry of 6-8 mm stages, according to [20].

| **Genotypes** | **Phenological**  **stage** | **Raw reads** | **Reads after trimming** | **Reads mapped** | |  |
| --- | --- | --- | --- | --- | --- | --- |
| SB_91 | Fruit setting | 7,784,105 | 7,706,202 | | 7,277,883 | |
| SB_151 | Fruit setting | 9,372,771 | 9,180,595 | | 8,691,987 | |
| SB_359 | Fruit setting | 12,941,624 | 12,740,412 | | 11,734,145 | |
| LB_19 | Fruit setting | 8,927,416 | 8,822,155 | | 8,261,160 | |
| LB_112 | Fruit setting | 10,885,400 | 10,728,881 | | 10,023,736 | |
| LB_117 | Fruit setting | 9,406,990 | 9,358,954 | | 8,768,223 | |
| Sultanina | Fruit setting | 10,483,239 | 10,306,135 | | 11,034,330 | |
| Ruby | Fruit setting | 11,703,667 | 11,581,925 | | 9,670,484 | |
| SB_91 | Berry of 6-8 mm | 5,560,072 | 5,498,863 | | 5,181,140 | |
| SB_359 | Berry of 6-8 mm | 4,758,771 | 4,682,808 | | 4,328,798 | |
| LB_19 | Berry of 6-8 mm | 11,425,990 | 11,291,471 | | 10,787,730 | |
| LB_112 | Berry of 6-8 mm | 23,640,636 | 23,263,080 | | 22,164,036 | |
| LB_117 | Berry of 6-8 mm | 18,573,815 | 18,295,075 | | 17,457,515 | |
| Sultanina | Berry of 6-8 mm | 9,596,386 | 9,440,741 | | 8,951,337 | |
