# Supplementary figures and images for: Identification of SNPs and InDels associated with berry size in table grapes integrating genetic and transcriptomic approaches
Source: BMC Plant Biol. 2020 Aug 3;20:365. doi: 10.1186/s12870-020-02564-4 (PMC7397606; doi:10.1186/s12870-020-02564-4)

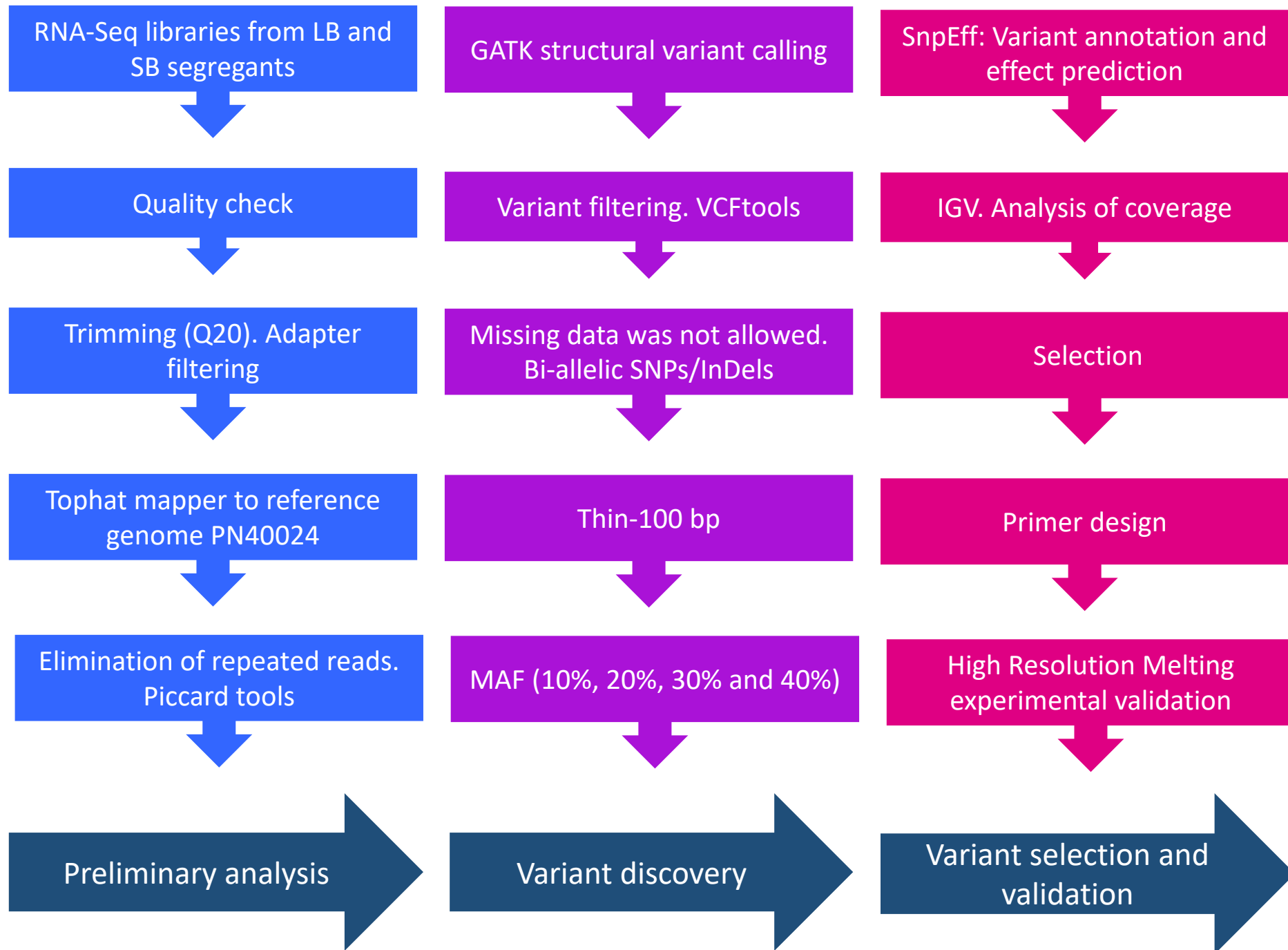

Supplement: Supplementary file 2 — Additional file 2: Figure S1. Bioinformatics workflow for the identification and characterization of SNP and InDel molecular markers derived from RNA-Seq data, associated with berry weight trait in Vitis vinifera. [file 12870_2020_2564_MOESM2_ESM.pdf]

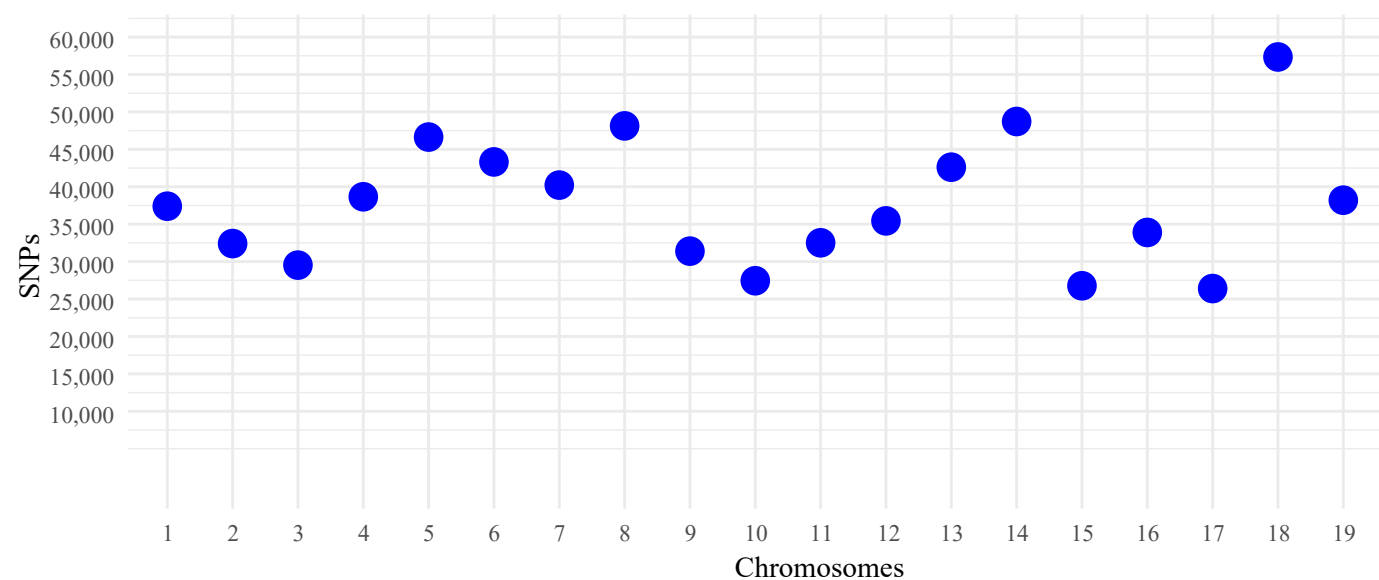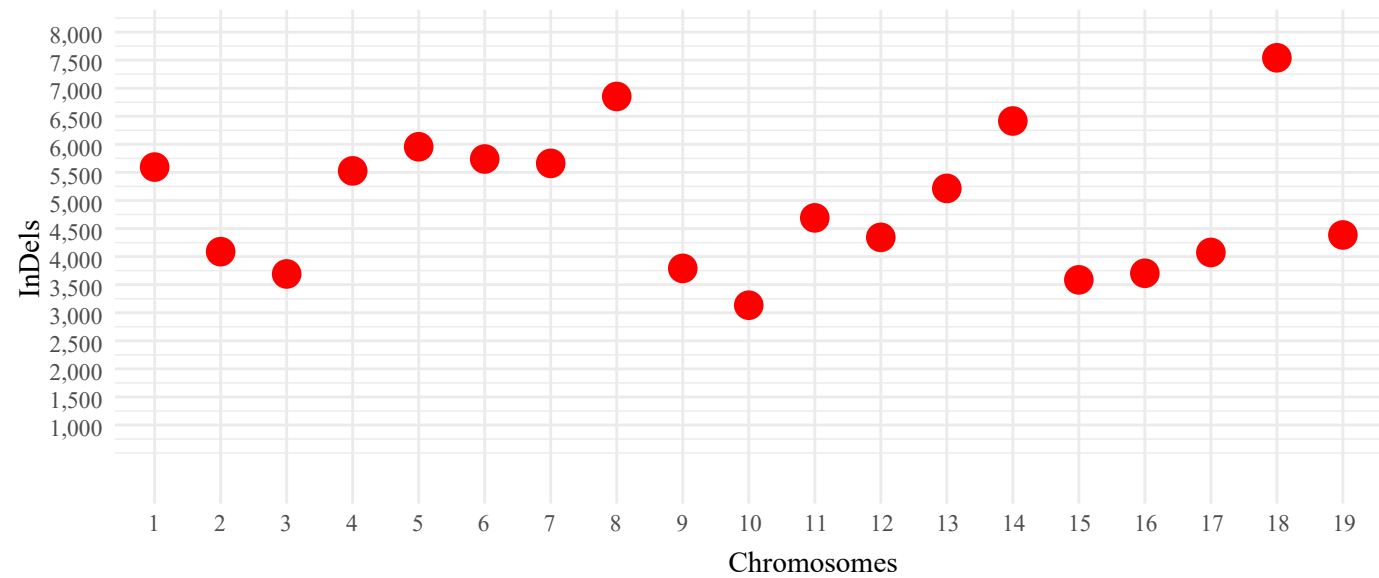

Supplement: Supplementary file 3 — Additional file 3: Figure S2A, S2B. Distribution of SNP (A) and InDel (B) polymorphisms along V. vinifera chromosomes. [file 12870_2020_2564_MOESM3_ESM.pdf]

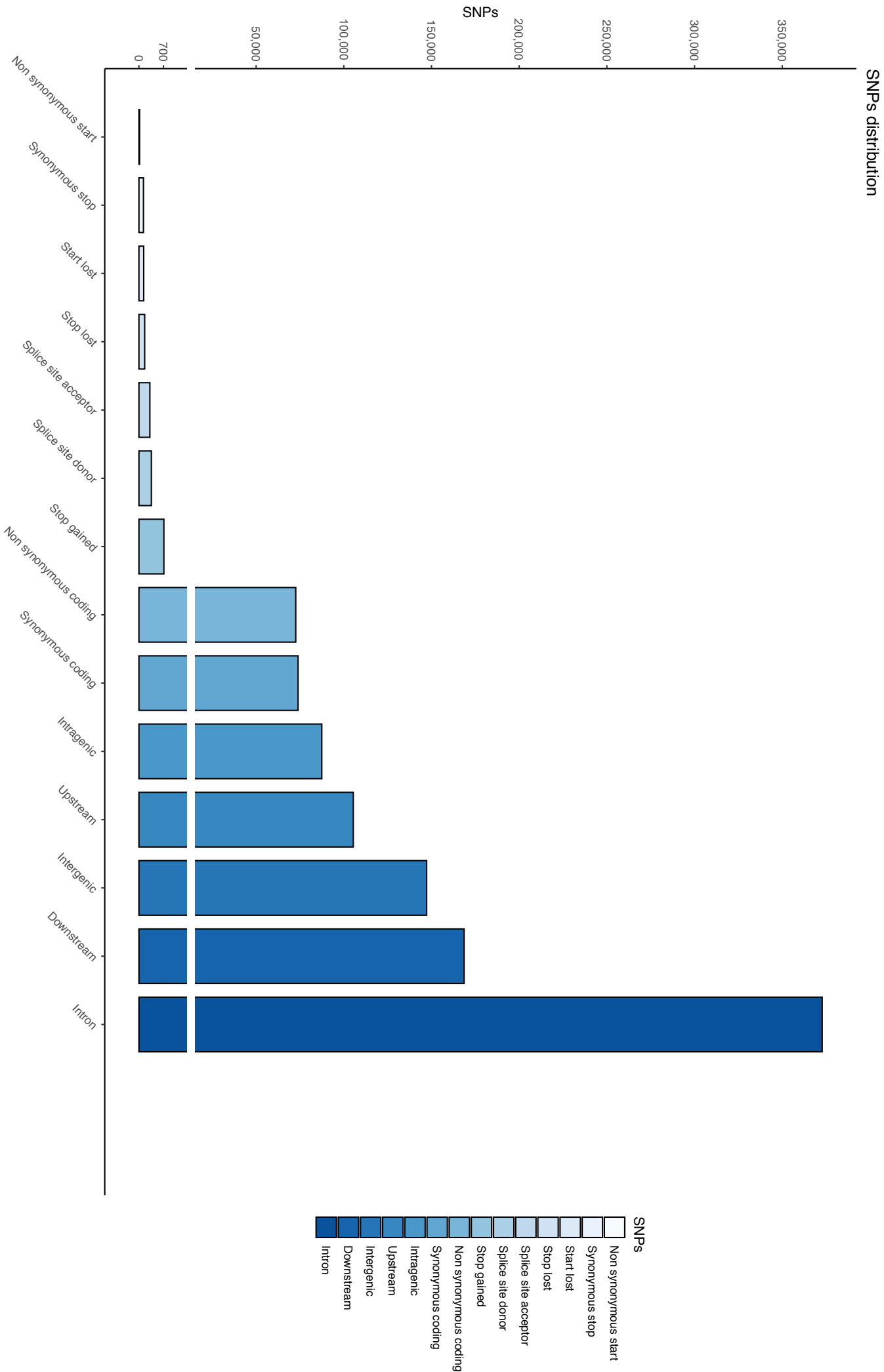

Supplement: Supplementary file 4 — Additional file 4: Figure S3. SNP distribution along the Vitis vinifera genome according to SnpEffect analysis based on a gene model for V. vinifera (PN40024). [file 12870_2020_2564_MOESM4_ESM.pdf]

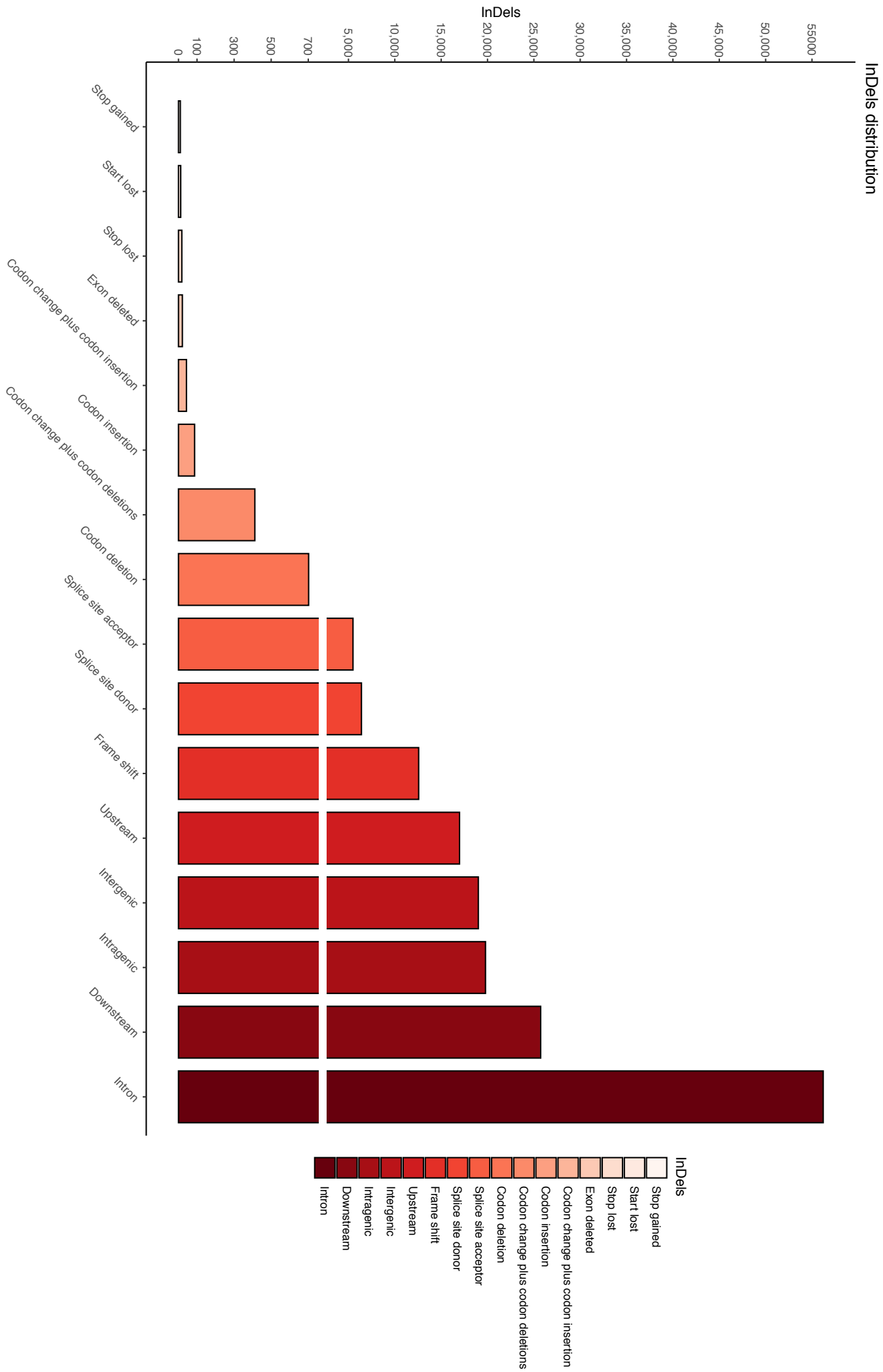

Supplement: Supplementary file 5 — Additional file 5: Figure S4. InDel distribution along the Vitis vinifera genome according to SnpEffect analysis based on a gene model for V. vinifera (PN40024). [file 12870_2020_2564_MOESM5_ESM.pdf]

SNP density (SNPs/Kb)

Chr

InDel density (InDels/Kb)

Un

9

8

7

6

5

4

3

2

19

18

17

16

15

14

13

12

11

10

1

2.0

1.5

1.0

0.5

0.0

0.0

0.1

0.2

0.3

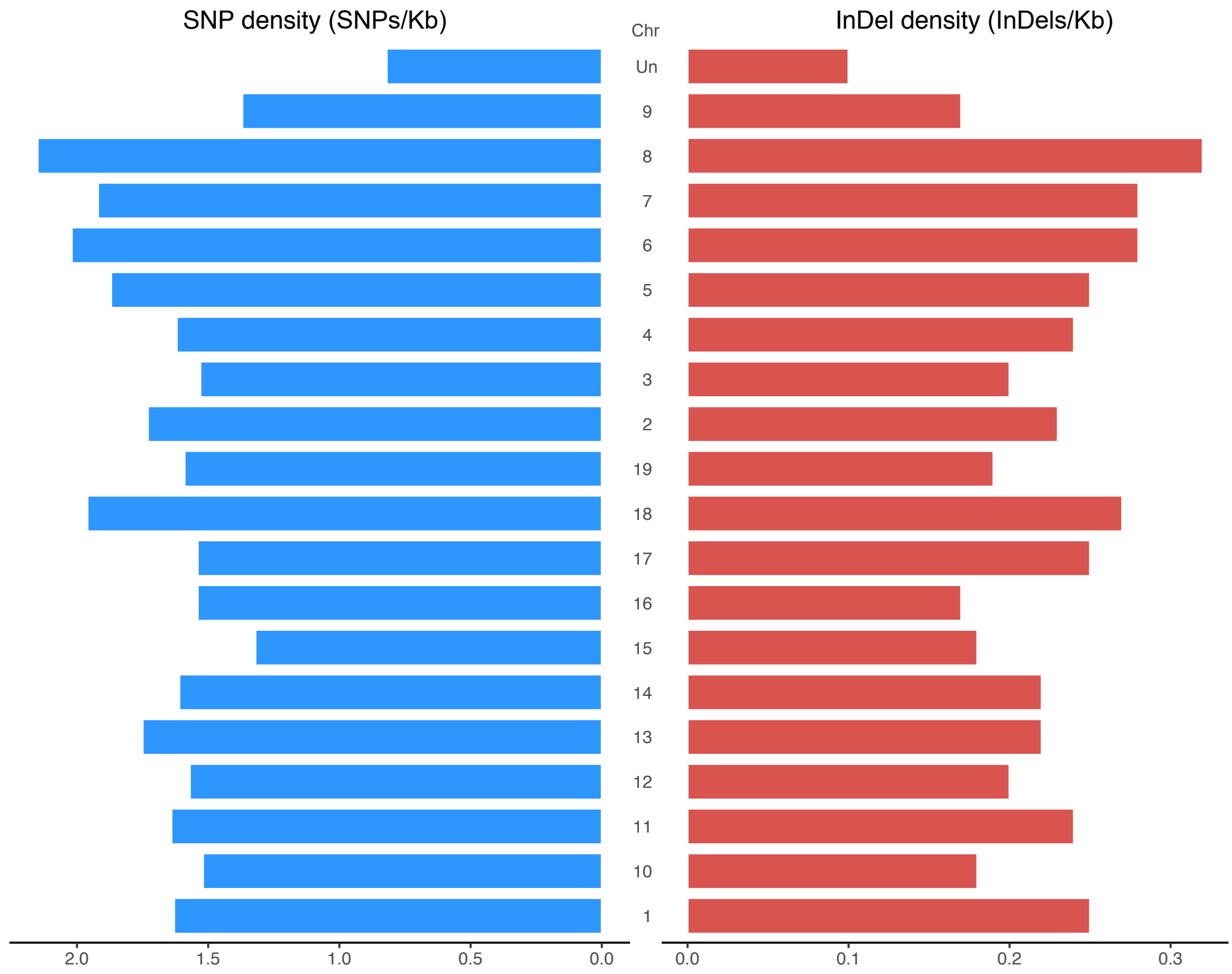

Supplement: Supplementary file 6 — Additional file 6: Figure S5. SNP and InDel density determined in V. vinifera chromosomes. Density was estimated considering the total observed polymorphisms per Kb. [file 12870_2020_2564_MOESM6_ESM.pdf]

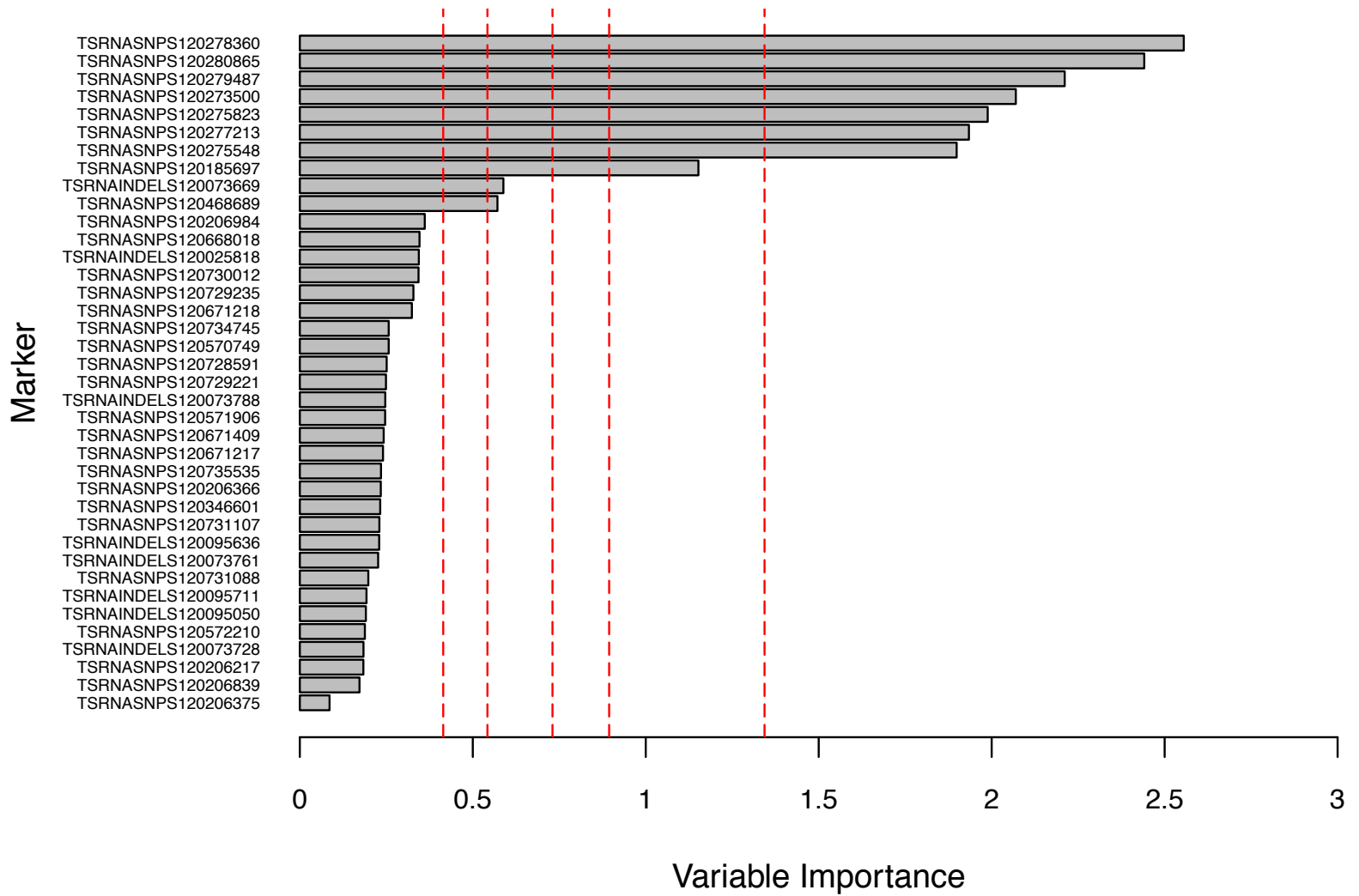

Supplement: Supplementary file 11 — Additional file 11: Figure S8. Random Forest regression analysis was applied to identify the most important SNP or/and InDel markers over the phenotype variation. Analysis was performed using observed genotypes of 38 candidate polymorphisms and the 13 segregants with known phenotype for berry weight, including the parents ‘Ruby seedless’ and ‘Sultanina’. Berry weight (BW) was considered as the response variable; 1000 permutations were used and decision trees were obtained, selecting a consensus. Red line indicates significant polymorphisms. At least 10 markers representing nine SNPs and one InDel were significantly associated with BW (p < 0.05). [file 12870_2020_2564_MOESM11_ESM.pdf]
