# Supplementary material for: Identification of SNPs and InDels associated with berry size in table grapes integrating genetic and transcriptomic approaches
Source: BMC Plant Biol. 2020 Aug 3;20:365. doi: 10.1186/s12870-020-02564-4 (PMC7397606; doi:10.1186/s12870-020-02564-4)
Supplement: Supplementary file 8 — Additional file 8: Table S2. Primers designed for the validation of 30 SNPs and the subsequent genotyping of seedless segregants from RxS population and table grape varieties, based on High Resolution Melting analysis (qPCR-HRM). [file 12870_2020_2564_MOESM8_ESM.docx]

**Supplementary Table S2.** Primers designed for the validation of 30 SNPs and the subsequent genotyping of seedless segregants from RxS crossing and table grape varieties, based on High Resolution Melting analysis (qPCR-HRM).

| **SNP_ID** | **Primer** | **Sequence (5'->3')** | **Tm (°C)** | **%GC** |
| --- | --- | --- | --- | --- |
| TSRNASNP120729221 | PF | GTGAGCCCAGGGATTATGAA | 60 | 50 |
|  | PR | TGAAGAACCAGCACGAACAG | 60 | 50 |
| TSRNASNP120731088 | PF | GGGAAGTTTAGCGAGGCTCT | 60 | 55 |
|  | PR | CACAGGTGAAAACTCCAGCA | 60 | 50 |
| TSRNASNP120735535 | PF | CTCCTGGTCAGGCTTCTCAG | 60 | 60 |
|  | PR | CCTGAATTTGAGGAGCAGGT | 59 | 50 |
| TSRNASNP120728591 | PF | GTAAAACGGATGCCCTTCAA | 60 | 45 |
|  | PR | CCGGTTGGGTTTCGATTTAT | 61 | 45 |
| TSRNASNP120729235 | PF | TTCTCGGGACTTCTCTTCCA | 60 | 50 |
|  | PR | GCTGGATGCAGAGCCACTAT | 60 | 55 |
| TSRNASNP120731107 | PF | GTCACCCGGTTAGGGACTCT | 60 | 60 |
|  | PR | TCAAATGCTCTCCAGGGACT | 60 | 50 |
| TSRNASNPS120185697 | PF | TAGAACGGAATCCGCAAATC | 60 | 45 |
|  | PR | GGGAAATTCCACCGTTATGA | 60 | 45 |
| TSRNASNPS120206217 | PF | ACAGGAGCTAATGGAACTGAG | 57 | 48 |
|  | PR | CCCCCTCTCATCTTACGCTT | 59 | 55 |
| TSRNASNPS120206366 | PF | GCTCAATCTGGGATTCAGGA | 60 | 50 |
|  | PR | TGGAAACTGGAGCAAACACA | 60 | 45 |
| TSRNASNPS120206375 | PF | GCAGTAAGTTATGAGGCACC | 56 | 50 |
|  | PR | GGGTTTCCACGTTGAGAAGA | 58 | 50 |
| TSRNASNPS120206839 | PF | CCTGGCTAAAGAAACAACCA | 58 | 45 |
|  | PR | TGCCACGTAATAAACGCAGA | 60 | 45 |
| TSRNASNPS120206984 | PF | GGGCTTTTGTGTACCCTTCA | 58 | 50 |
|  | PR | CCTGTACTCTCTTCTTTTCCAC | 56 | 45 |
| TSRNASNPS120273500 | PF | TTGTGGCAGTGGTTGGACA | 60 | 53 |
|  | PR | CCATTGATGCAGCGACACT | 59 | 53 |
| TSRNASNPS120275548 | PF | GCAAGAAAAGCATGAGACCA | 59 | 45 |
|  | PR | TCAAACATCCAACAGCAAGC | 60 | 45 |
| TSRNASNPS120275823 | PF | ACCTGCTTTACTCTCTCCACT | 58 | 48 |
|  | PR | GGAAGAAAACATCATCAAGGGT | 57 | 41 |
| TSRNASNPS120277213 | PF | GGGACATCAGTTGCTTAACAGT | 59 | 45 |
|  | PR | TAGCCTCCATCTTGACACAG | 57 | 50 |
| TSRNASNPS120278360 | PF | TGGGGGCAAAACTAAGAAAA | 60 | 40 |
|  | PR | ACATGAGCGGGAAGATGAAG | 60 | 50 |
| TSRNASNPS120279487 | PF | TCACCCTTCTCGGCCTTT | 58 | 56 |
|  | PR | ACAGCGACTAAAACTAGAACAC | 56 | 41 |
| TSRNASNPS120280865 | PF | CCTCTGATAAAGGCAAAACCA | 56 | 43 |
|  | PR | TAGACAGTTGTATGCTCCTGC | 57 | 48 |
| TSRNASNPS120346601 | PF | GATGCCAAGAAGCAGCAATC | 61 | 50 |
|  | PR | TGACGCCTAAATCCCATGAT | 60 | 45 |
| TSRNASNPS120468689 | PF | TTCATACCCACCAAATTCAGG | 60 | 42 |
|  | PR | AAAAGGAAATCACCTGGAGGA | 60 | 42 |
| TSRNASNPS120570749 | PF | TCCATGGCATTCCCTTATGT | 60 | 45 |
|  | PR | GAGCATGACTCCAGGAAAGC | 60 | 55 |
| TSRNASNPS120571906 | PF | AACACAAGCCGACTGACCA | 59 | 53 |
|  | PR | CAGACGCTACCACCTTCCAC | 60 | 60 |
| TSRNASNPS120572210 | PF | TTTCAAGATCAGGTAGTGCGTT | 58 | 41 |
|  | PR | AAACAAGTATGCAGGGGGTCA | 60 | 48 |
| TSRNASNPS120668018 | PF | ACACGCTGTTCTTCAACCAG | 59 | 50 |
|  | PR | GGAAAAAGGCCGAGGAAT | 59 | 50 |
| TSRNASNPS120671217 | PF | CAGCGAAAGTGTGACGAAAC | 59 | 50 |
|  | PR | TTGAAAATGCGTTGAGGACT | 58 | 40 |
| TSRNASNPS120671218 | PF | TGCCTAAAAATTGAAGTGCGGA | 59 | 41 |
|  | PR | CCTTATCCCCCTTGCTCTGC | 60 | 60 |
| TSRNASNPS120671409 | PF | GCATGCTGCTTCTCTGGAAT | 61 | 50 |
|  | PR | CTGCATGCTGCTCCTACTTG | 60 | 55 |
| TSRNASNPS120730012 | PF | AAATGGCTTTGCCCGTGG | 59 | 56 |
|  | PR | GAGGAACACAAGGAGGATGG | 58 | 55 |
| TSRNASNPS120734745 | PF | GCTGCTACTGAAACTACCGA | 57 | 50 |
|  | PR | GCTTGGAATACCTACAAATGCT | 57 | 41 |

PF= Primer forward, PR= Primer reverse.
