# Supplementary material for: Identification of SNPs and InDels associated with berry size in table grapes integrating genetic and transcriptomic approaches
Source: BMC Plant Biol. 2020 Aug 3;20:365. doi: 10.1186/s12870-020-02564-4 (PMC7397606; doi:10.1186/s12870-020-02564-4)
Supplement: Supplementary file 9 — Additional file 9: Table S3. Primers designed for the validation of eight InDels and the subsequent genotyping of seedless segregants from RxS crossing and table grape varieties, based on High Resolution Melting analysis (qPCR-HRM). [file 12870_2020_2564_MOESM9_ESM.docx]

**Supplementary Table S3.** Primers designed for the validation of eight InDels and the subsequent genotyping of seedless segregants from RxS crossing and table grape varieties, based on High Resolution Melting analysis (qPCR-HRM).

| **InDel_ID** | **Primer** | **Sequence (5'->3')** | **Tm** | **%GC** |
| --- | --- | --- | --- | --- |
| TSRNAINDELS120025818 | PF | GATGGACCCTGGAGAGACAA | 60 | 55 |
|  | PR | CAGAGAAAGGGAACCGAAGA | 59 | 50 |
| TSRNAINDELS120073669 | PF | TGAGAGGAGCCTGAGGGTTA | 60 | 55 |
|  | PR | AGAGATGAGAGAGGCCACCA | 60 | 55 |
| TSRNAINDELS120073728 | PF | CAACCTCACACACCCTCAAA | 60 | 50 |
|  | PR | GGCACTGATCACACAAAACG | 60 | 50 |
| TSRNAINDELS120073761 | PF | TGAAACCCCATATCCGAAAA | 60 | 40 |
|  | PR | TTGTGTATTGGCTGCAGAGC | 60 | 50 |
| TSRNAINDELS120073788 | PF | CGCCGTTCATCCACACTACT | 60 | 55 |
|  | PR | GAAGCCTTGCTGGGACAATA | 59 | 50 |
| TSRNAINDELS120095050 | PF | AAGCGTGCAACTCACTGATG | 60 | 50 |
|  | PR | ATCCTTGGGGAAATGGATTC | 57 | 45 |
| TSRNAINDELS120095636 | PF | GAAGAGCGAAGATCGGTGTC | 59 | 55 |
|  | PR | CAGAAACTTCCACCCAGCAT | 60 | 50 |
| TSRNAINDELS120095711 | PF | TTCTCCAATCTTTGGCATCA | 57 | 40 |
|  | PR | GAGGCAGGGTATGCTTTGAG | 61 | 55 |
|  |  |  |  |  |

PF= Primer forward, PR= Primer reverse.
