# Supplementary material for: Identification of SNPs and InDels associated with berry size in table grapes integrating genetic and transcriptomic approaches
Source: BMC Plant Biol. 2020 Aug 3;20:365. doi: 10.1186/s12870-020-02564-4 (PMC7397606; doi:10.1186/s12870-020-02564-4)
Supplement: Supplementary file 10 — Additional file 10: Fig. S7. Hierarchical tree summarizing over-represented gene ontology (GO) categories, identified in a set of 32 genes containing SNP and InDel markers. Dots at branches represent significant FDR values. [file 12870_2020_2564_MOESM10_ESM.docx]

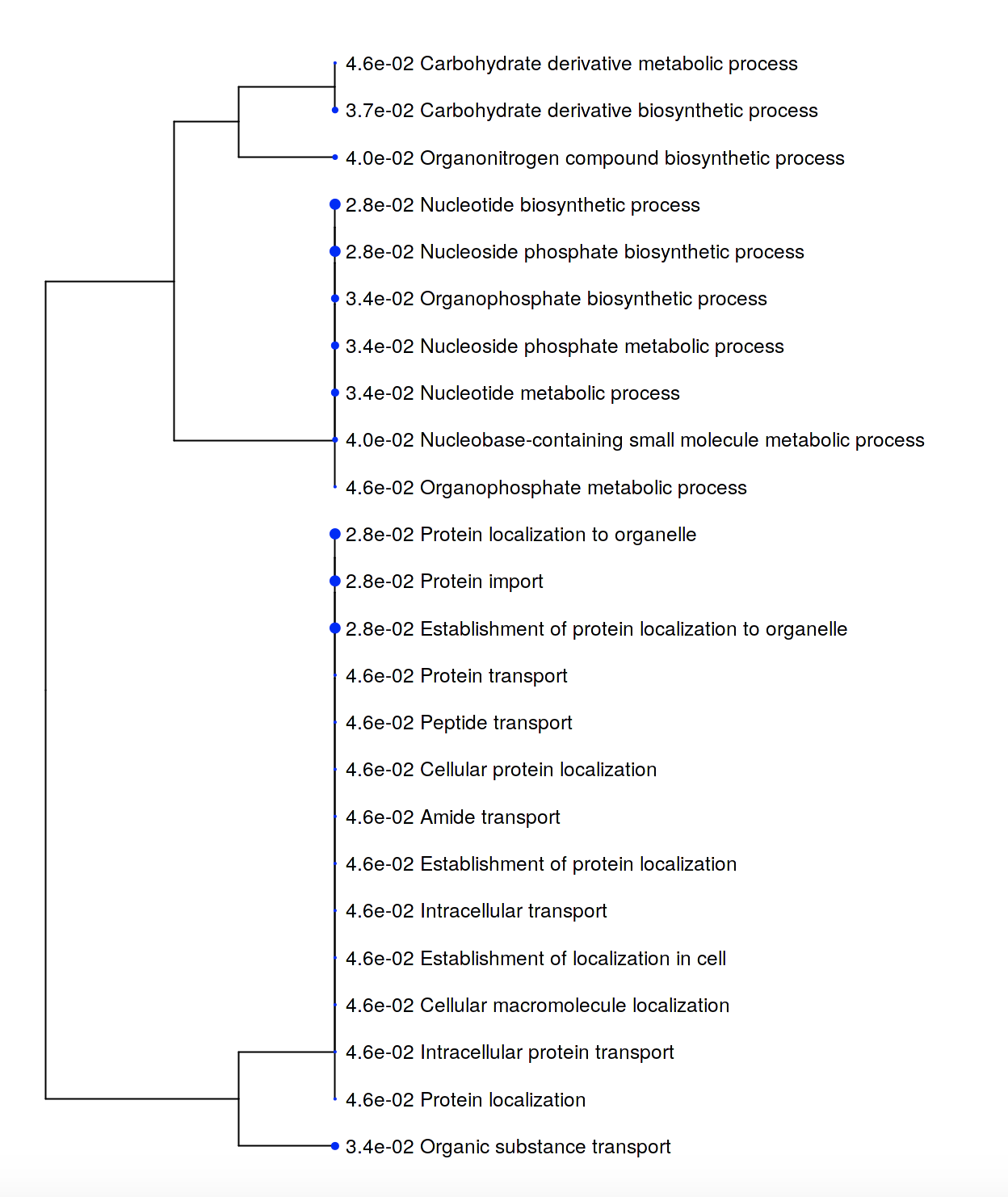


Localization

Biosynthetic process

Supplementary Figure S7. Hierarchical tree summarizing over-represented gene ontology (GO) categories, identified in a set of 32 genes containing SNP and InDel markers. Dots at branches represent significant FDR values
