# Supplementary material for: Identification of SNPs and InDels associated with berry size in table grapes integrating genetic and transcriptomic approaches
Source: BMC Plant Biol. 2020 Aug 3;20:365. doi: 10.1186/s12870-020-02564-4 (PMC7397606; doi:10.1186/s12870-020-02564-4)
Supplement: Supplementary file 12 — Additional file 12: Table S4. Unbalanced analysis of variance (ANOVA) to analyze possible effects of 30 SNP and eight InDels candidates associated with berry weight. [file 12870_2020_2564_MOESM12_ESM.docx]

**Supplementary Table S4**. Unbalanced analysis of variance (ANOVA) to analyze possible effect of 30 SNPs and eight InDels markers associated with berry weight.

| **Marker** | **Type** | **R^2^** | **p-value** | **Chr** |
| --- | --- | --- | --- | --- |
| TSRNASNPS120279487 | SNP | 0.325 | 3.59E-11 | 17 |
| TSRNASNPS120277213 | SNP | 0.325 | 3.59E-11 | 17 |
| TSRNASNPS120280865 | SNP | 0.325 | 3.59E-11 | 17 |
| TSRNASNPS120275823 | SNP | 0.325 | 3.59E-11 | 17 |
| TSRNASNPS120273500 | SNP | 0.325 | 3.59E-11 | 17 |
| TSRNASNPS120278360 | SNP | 0.271 | 2.89E-09 | 17 |
| TSRNAINDELS120025818 | InDel | 0.186 | 1.07E-05 | 15 |
| TSRNASNPS120206984 | SNP | 0.186 | 1.07E-05 | 15 |
| TSRNASNPS120275548 | SNP | 0.179 | 2.77E-06 | 17 |
| TSRNAINDELS120073669 | InDel | 0.135 | 5.79E-05 | 6 |
| TSRNASNPS120728591 | SNP | 0.089 | 1.31E-03 | 9 |
| TSRNASNPS120731088 | SNP | 0.089 | 1.31E-03 | 9 |
| TSRNAINDELS120095636 | InDel | 0.089 | 1.31E-03 | 9 |
| TSRNASNPS120731107 | SNP | 0.089 | 1.31E-03 | 9 |
| TSRNASNPS120734745 | SNP | 0.089 | 1.31E-03 | 9 |
| TSRNASNPS120730012 | SNP | 0.089 | 1.31E-03 | 9 |
| TSRNASNPS120735535 | SNP | 0.089 | 1.31E-03 | 9 |
| TSRNASNPS120729221 | SNP | 0.089 | 1.31E-03 | 9 |
| TSRNAINDELS120095050 | InDel | 0.089 | 1.31E-03 | 9 |
| TSRNAINDELS120095711 | InDel | 0.089 | 1.31E-03 | 9 |
| TSRNASNPS120729235 | SNP | 0.089 | 1.31E-03 | 9 |
| TSRNASNPS120671218 | SNP | 0.083 | 1.89E-03 | 8 |
| TSRNASNPS120671217 | SNP | 0.083 | 1.89E-03 | 8 |
| TSRNASNPS120668018 | SNP | 0.083 | 1.89E-03 | 8 |
| TSRNASNPS120671409 | SNP | 0.083 | 1.89E-03 | 8 |
| TSRNAINDELS120073728 | InDel | 0.072 | 3.98E-03 | 6 |
| TSRNASNPS120571906 | SNP | 0.072 | 3.98E-03 | 6 |
| TSRNASNPS120570749 | SNP | 0.072 | 3.98E-03 | 6 |
| TSRNAINDELS120073788 | InDel | 0.072 | 3.98E-03 | 6 |
| TSRNAINDELS120073761 | InDel | 0.072 | 3.98E-03 | 6 |
| TSRNASNPS120572210 | SNP | 0.072 | 3.98E-03 | 6 |
| TSRNASNPS120346601 | SNP | 0.066 | 5.72E-03 | 19 |
| TSRNASNPS120468689 | SNP | 0.032 | 5.53E-02 | 3 |
| TSRNASNPS120185697 | SNP | 0.026 | 8.90E-02 | 14 |
| TSRNASNPS120206375 | SNP | 0.014 | 2.04E-01 | 15 |
| TSRNASNPS120205444 | SNP | 0.000 | 8.62E-01 | 15 |
| TSRNASNPS120206839 | SNP | 0.000 | 8.62E-01 | 15 |
| TSRNASNPS120206366 | SNP | 0.000 | 8.62E-01 | 15 |
