# Supplementary material for: Identification of SNPs and InDels associated with berry size in table grapes integrating genetic and transcriptomic approaches
Source: BMC Plant Biol. 2020 Aug 3;20:365. doi: 10.1186/s12870-020-02564-4 (PMC7397606; doi:10.1186/s12870-020-02564-4)
Supplement: Supplementary file 13 — Additional file 13: Table S5. Collection of 21 table grape varieties, representative of table grape diversity cultivated in Chile. [file 12870_2020_2564_MOESM13_ESM.docx]

**Supplementary Table S5.** Collection of 21 table grapes varieties, representative of table grapes diversity cultivated in Chile.

| **Variety** | **Country** |
| --- | --- |
| Autumn Royal | USA |
| Autumn seedless | USA |
| Beauty seedless | USA |
| Big Red | USA |
| Black seedless | Chile |
| Blush seedless | USA |
| Calmeria | USA |
| Christmas Rose | USA |
| Crimson seedless | USA |
| Dawn seedless | USA |
| Emperor | USA |
| Flame seedless | USA |
| Ilusión | Chile |
| Italia Pirovano | Italia |
| Melissa | USA |
| Perlette | USA |
| Perlón | Argentina |
| Red seedless | USA |
| Ruby seedless | USA |
| Sultanina | Turkey |
| Superior seedless | USA |
