# Supplementary material for: Identification of SNPs and InDels associated with berry size in table grapes integrating genetic and transcriptomic approaches
Source: BMC Plant Biol. 2020 Aug 3;20:365. doi: 10.1186/s12870-020-02564-4 (PMC7397606; doi:10.1186/s12870-020-02564-4)
Supplement: Supplementary file 14 — Additional file 14: Table S6. International collection of 20 varieties, representative of genetic diversity of Vitis vinifera Prole ‘Orientalis’. [file 12870_2020_2564_MOESM14_ESM.docx]

**Supplementary Table S6.** International collection of 20 varieties, representative of genetic diversity of *Vitis vinifera* Prole ‘*Orientalis’.*

| **Variety name** | **Country** |
| --- | --- |
| Ak ouzioum tagapskii | Kyrgyzstan |
| Araklinos | Greece |
| Assyl kara | Russia |
| Cabernet franc | France |
| César | France |
| Chirai obak | Tajikistan |
| Chouchillon | France |
| Espadeiro tinto | Portugal |
| Kapistroni tétri hermaphrodite (Coll. Kichinev) | Georgia |
| Lameiro | Portugal |
| Médouar | Israel |
| Mehdik | Iran |
| Orbois | France |
| Orlovi nokti | Russia |
| Pervenetz praskoveïsky | Russia |
| Plant du Maroc E (Coll. Meknès) | Morocco |
| Pletchistik | Russia (near Rostov) |
| Tsitsa Kaprei | Moldavie |
| Tsolikouri | Georgia |
| Variété d’oasis Bou Chemma 46 | Tunisia |
