# Supplementary material for: Identification of SNPs and InDels associated with berry size in table grapes integrating genetic and transcriptomic approaches
Source: BMC Plant Biol. 2020 Aug 3;20:365. doi: 10.1186/s12870-020-02564-4 (PMC7397606; doi:10.1186/s12870-020-02564-4)
Supplement: Supplementary file 15 — Additional file 15: Table S7. Co-localization of 38 SNP/InDel markers associated with berry weight, and differentially expressed genes (DE genes) between large and small berry segregants, previously reported by [20]. The total of DE genes located in genomic regions overlapping physical coordinates of SNP/InDel markers is shown, encompassing eight chromosomes. DE genes significantly correlated with PCA-1 component, are highlighted in yellow. [file 12870_2020_2564_MOESM15_ESM.docx]

**Supplementary Table S7**. Co-localization of 38 SNP/InDel markers associated with berry weight, and differentially expressed genes (DE genes) between large and small berry segregants, previously reported by [20]. The total of DE genes located in genomic regions overlapping physical coordinates of SNP/InDel markers is shown, encompassing eight chromosomes. DE genes significantly correlated with PCA-1 component, are highlighted in yellow.

| **Marker** | **Chr** | **DE genes** | **Gene_ID** | **Description** |
| --- | --- | --- | --- | --- |
| SNPs | 3 | 25 | Vitvi03g00717 | Egy3 egy3 (ethylene-dependent gravitropism-deficient and yellow-green-like 3) |
|  |  |  | Vitvi03g00703 | Eugenol synthase 1 |
|  |  |  | Vitvi03g00209 | Expansin-like A2 |
|  |  |  | Vitvi03g00708 | Serine carboxypeptidase-like 18 |
|  |  |  | Vitvi03g00306 | F-box At2g16365 protein |
|  |  |  | Vitvi03g01288 | Zinc transporter 8 |
|  |  |  | Vitvi03g00500 | Alpha-amylase type B isozyme |
|  |  |  | Vitvi03g00745 | Basic form of pathogenesis-related protein 1 |
|  |  |  | Vitvi03g01597 | putative secretory protein |
|  |  |  | Vitvi03g00334 | 2-hydroxyacid dehydrogenase putative |
|  |  |  | Vitvi03g00023 | DNA replication licensing factor mcm4 |
|  |  |  | Vitvi03g00742 | Basic form of pathogenesis-related protein 1 |
|  |  |  | Vitvi03g00509 | Ferredoxin--nitrite reductase chloroplastic |
|  |  |  | Vitvi03g01483 | AMP-activated protein kinase gamma regulatory subunit putative |
|  |  |  | Vitvi03g01571 | Alpha-amylase |
|  |  |  | Vitvi03g00712 | Phytosulfokine |
|  |  |  | Vitvi03g01833 | ABC transporter G family member 11 |
|  |  |  | Vitvi03g00315 | Transcription factor bHLH63 |
|  |  |  | Vitvi03g00080 | calcium ion binding protein putative |
|  |  |  | Vitvi03g00586 | Probable indole-3-acetic acid-amido synthetase GH3.1 |
|  |  |  | Vitvi03g00122 | Auxin transporter-like protein 3 |
|  |  |  | Vitvi03g00213 | Lysine histidine transporter-like 8 |
|  |  |  | Vitvi03g00124 | Pathogenesis-related protein 5 |
|  |  |  | Vitvi03g00700 | Eugenol synthase 1 |
|  |  |  | Vitvi03g00102 | Unknown Protein Function |
|  |  |  |  |  |
| SNPs/InDels | 6 | 26 | Vitvi06g00790 | Expansin-A8 |
|  |  |  | Vitvi06g00582 | Uncharacterized RNA-binding protein C25G10.01 |
|  |  |  | Vitvi06g00679 | GCN5-related N-acetyltransferase (GNAT) family protein |
|  |  |  | Vitvi06g00613 | similar to binding |
|  |  |  | Vitvi06g00443 | Heat shock cognate 70 kDa protein 2 |
|  |  |  | Vitvi06g00576 | Proliferating cell nuclear antigen |
|  |  |  | Vitvi06g00667 | Probable protein phosphatase 2C 25 |
|  |  |  | Vitvi06g01917 | Lichenase |
|  |  |  | Vitvi06g00784 | Probable serine/threonine-protein kinase WNK4 |
|  |  |  | Vitvi06g01629 | Unknown Protein Function |
|  |  |  | Vitvi06g00368 | Two-component response regulator-like PRR73 |
|  |  |  | Vitvi06g01601 | Unknown Protein Function |
|  |  |  | Vitvi06g01459 | Cucumisin |
|  |  |  | Vitvi06g00656 | Omega-3 fatty acid desaturase chloroplastic |
|  |  |  | Vitvi06g01471 | Unknown Protein Function |
|  |  |  | Vitvi06g01630 | Unknown Protein Function |
|  |  |  | Vitvi06g01746 | 4-hydroxyphenylacetaldehyde oxime monooxygenase |
|  |  |  | Vitvi06g01340 | Unknown Protein Function |
|  |  |  | Vitvi06g01718 | Tropinone reductase homolog At1g07440 |
|  |  |  | Vitvi06g01605 | Unknown Protein Function |
|  |  |  | Vitvi06g00920 | Uncharacterized basic helix-loop-helix protein At1g64625 |
|  |  |  | Vitvi06g00529 | Tropinone reductase homolog At1g07440 |
|  |  |  | Vitvi06g00984 | Probable gibberellin receptor GID1L3 |
|  |  |  | Vitvi06g01331 | Vacuolar amino acid transporter 1 |
|  |  |  | Vitvi06g00771 | Peroxidase 4 |
|  |  |  | Vitvi06g01714 | Tropinone reductase homolog At1g07440 |
|  |  |  |  |  |
| SNPs | 8 | 25 | Vitvi08g01196 | Metallothionein-like protein type 2 |
|  |  |  | Vitvi08g00822 | Cationic peroxidase 1 |
|  |  |  | Vitvi08g02225 | Unknown Protein Function |
|  |  |  | Vitvi08g00794 | F-box/LRR-repeat protein 3 |
|  |  |  | Vitvi08g02189 | Unknown Protein Function |
|  |  |  | Vitvi08g01750 | Peroxidase 53 |
|  |  |  | Vitvi08g01217 | Uncharacterized protein C24B11.05 |
|  |  |  | Vitvi08g01801 | Putative mitochondrial 2-oxoglutarate/malate carrier protein |
|  |  |  | Vitvi08g02007 | F-box/LRR-repeat protein At3g48880 |
|  |  |  | Vitvi08g01623 | Transcription factor RAX2 |
|  |  |  | Vitvi08g01370 | Probable receptor protein kinase TMK1 |
|  |  |  | Vitvi08g01856 | Transcription factor bHLH60 |
|  |  |  | Vitvi08g00802 | Wound-induced protein putative |
|  |  |  | Vitvi08g01701 | Glucan endo-13-beta-glucosidase |
|  |  |  | Vitvi08g00957 | Probable glutathione S-transferase |
|  |  |  | Vitvi08g01757 | Activator of 90 kDa heat shock protein ATPase homolog |
|  |  |  | Vitvi08g01702 | Glucan endo-13-beta-glucosidase basic isoform |
|  |  |  | Vitvi08g00723 | Ammonium transporter 3 member 3 |
|  |  |  | Vitvi08g01384 | Aspartic proteinase nepenthesin-2 |
|  |  |  | Vitvi08g02231 | Fructose-16-bisphosphatase chloroplastic |
|  |  |  | Vitvi08g01033 | Auxin response factor 8 |
|  |  |  | Vitvi08g01637 | Flavonoid 3'5'-hydroxylase |
|  |  |  | Vitvi08g02189 | Heat shock cognate 70 kDa protein 2 |
|  |  |  | Vitvi08g01055 | MLO-like protein 12 |
|  |  |  | Vitvi08g02228 | Probable glutathione S-transferase |
|  |  |  |  |  |
| SNPs/InDels | 9 | 25 | Vitvi09g00377 | Mitochondrial carnitine/acylcarnitine carrier protein CACL |
|  |  |  | Vitvi09g00183 | Protein HOTHEAD |
|  |  |  | Vitvi09g00281 | Auxin-responsive protein IAA26 |
|  |  |  | Vitvi09g01604 | Putative Peroxidase 48 |
|  |  |  | Vitvi09g00436 | Auxin-induced protein AUX22 |
|  |  |  | Vitvi09g00517 | (6-4)DNA photolyase |
|  |  |  | Vitvi09g00184 | Serine/threonine-protein kinase PBS1 |
|  |  |  | Vitvi09g01398 | Probable mitochondrial chaperone bcs1 |
|  |  |  | Vitvi09g00907 | Omega-hydroxypalmitate O-feruloyl transferase |
|  |  |  | Vitvi09g00512 | Unknown Protein Function |
|  |  |  | Vitvi09g01429 | Lupeol synthase 5 |
|  |  |  | Vitvi09g01413 | Camelliol C synthase |
|  |  |  | Vitvi09g00045 | 18.6 kDa class III heat shock protein |
|  |  |  | Vitvi09g00049 | Chaperone protein dnaJ |
|  |  |  | Vitvi09g01229 | Anthranilate N-benzoyltransferase protein 2 |
|  |  |  | Vitvi09g01164 | Chaperone protein dnaJ 8 chloroplastic |
|  |  |  | Vitvi09g01553 | Unknown Protein Function |
|  |  |  | Vitvi09g01944 | 60S ribosomal protein L7-4 |
|  |  |  | Vitvi09g00500 | Probable boron transporter 2 |
|  |  |  | Vitvi09g00303 | Pleiotropic drug resistance protein 15 |
|  |  |  | Vitvi09g02003 | Lupeol synthase 5 |
|  |  |  | Vitvi09g01664 | Cucumber peeling cupredoxin |
|  |  |  | Vitvi09g01887 | Protein WAX2 |
|  |  |  | Vitvi09g00350 | gb\|AAO63445.1\| At2g25737 |
|  |  |  | Vitvi09g01933 | 60S ribosomal protein L7-3 |
|  |  |  |  |  |
| SNPs | 14 | 28 | Vitvi14g02467 | Unknown Protein Function |
|  |  |  | Vitvi14g01967 | Os09g0413600 |
|  |  |  | Vitvi14g00322 | Probable gibberellin receptor GID1L1 |
|  |  |  | Vitvi14g01946 | Vacuolar amino acid transporter 1 |
|  |  |  | Vitvi14g01973 | Dof zinc finger protein DOF5.2 |
|  |  |  | Vitvi14g01717 | Galactinol--sucrose galactosyltransferase |
|  |  |  | Vitvi14g01336 | pollen Ole e 1 allergen and extensin family protein |
|  |  |  | Vitvi14g02542 | Germin-like protein subfamily 1 member 13 |
|  |  |  | Vitvi14g02017 | Phosphatidylinositol-345-trisphosphate 3-phosphatase and dual-specificity protein phosphatase PTEN |
|  |  |  | Vitvi14g00270 | Copper chaperone |
|  |  |  | Vitvi14g01951 | Vacuolar amino acid transporter 1 |
|  |  |  | Vitvi14g00339 | Bifunctional 3-dehydroquinate dehydratase/shikimate dehydrogenase chloroplastic |
|  |  |  | Vitvi14g02533 | Germin-like protein subfamily 1 member 15 |
|  |  |  | Vitvi14g02013 | hypothetical protein LOC100261075 putative transcriptional regulator |
|  |  |  | Vitvi14g02028 | Probable galacturonosyltransferase 13 |
|  |  |  | Vitvi14g00223 | Germin-like protein subfamily 1 member 15 |
|  |  |  | Vitvi14g00220 | Germin-like protein subfamily 1 member 15 |
|  |  |  | Vitvi14g02535 | Germin-like protein subfamily 1 member 15 |
|  |  |  | Vitvi14g01529 | nodulin family protein |
|  |  |  | Vitvi14g03084 | Gibberellin-regulated protein 4 |
|  |  |  | Vitvi14g00487 | Pathogenesis-related protein PR-4B |
|  |  |  | Vitvi14g00221 | Germin-like protein subfamily 1 member 15 |
|  |  |  | Vitvi14g02536 | Germin-like protein subfamily 1 member 15 |
|  |  |  | Vitvi14g01269 | Vacuolar cation/proton exchanger 2 |
|  |  |  | Vitvi14g00037 | nucleic acid binding protein putative |
|  |  |  | Vitvi14g01351 | Cytochrome P450 85A1 |
|  |  |  | Vitvi14g00269 | Basic 7S globulin |
|  |  |  | Vitvi14g00468 | Calmodulin-related protein |
|  |  |  |  |  |
| SNPs/InDels | 15 | 15 | Vitvi15g00938 | Myb-related protein Zm38 |
|  |  |  | Vitvi15g00962 | O-acyltransferase WSD1 |
|  |  |  | Vitvi15g00961 | Unknown Protein Function |
|  |  |  | Vitvi15g01512 | Cytochrome P450 76C2 |
|  |  |  | Vitvi15g01084 | Late embryogenesis abundant protein Lea14-A |
|  |  |  | Vitvi15g01151 | KTEL motif-containing protein 1 |
|  |  |  | Vitvi15g00396 | Isoflavone-7-O-methyltransferase 6 |
|  |  |  | Vitvi15g01049 | Fatty acid desaturase 3 |
|  |  |  | Vitvi15g00959 | O-acyltransferase WSD1 |
|  |  |  | Vitvi15g00787 | Gibberellin receptor GID1 |
|  |  |  | Vitvi15g00835 | Cytochrome P450 87A3 |
|  |  |  | Vitvi15g00879 | Two-component response regulator-like APRR5 |
|  |  |  | Vitvi15g01503 | Dihydroflavonol-4-reductase |
|  |  |  | Vitvi15g00399 | Protein WAX2 |
|  |  |  | Vitvi15g00765 | Transcription factor TCP9 |
|  |  |  |  |  |
| SNPs | 17 | 25 | Vitvi17g01251 | Expansin-A15 |
|  |  |  | Vitvi17g00173 | Nudix hydrolase 17 mitochondrial |
|  |  |  | Vitvi17g01497 | UDP-glucoronosyl and UDP-glucosyl transferase |
|  |  |  | Vitvi17g01451 | Germin-like protein subfamily T member 1 |
|  |  |  | Vitvi17g01368 | integral membrane protein DUF6 containing protein |
|  |  |  | Vitvi17g00070 | Protein RUPTURED POLLEN GRAIN 1 |
|  |  |  | Vitvi17g00540 | Homeobox-leucine zipper protein HOX16 |
|  |  |  | Vitvi17g00232 | Transcription factor MYB3 |
|  |  |  | Vitvi17g00658 | C2H2-type zinc finger transcription factor |
|  |  |  | Vitvi17g00333 | Predicted membrane protein |
|  |  |  | Vitvi17g00615 | Chlorophyll a-b binding protein 4 chloroplastic |
|  |  |  | Vitvi17g01557 | DEAD-box ATP-dependent RNA helicase 30 |
|  |  |  | Vitvi17g00445 | DNA polymerase epsilon subunit 2 |
|  |  |  | Vitvi17g00695 | Chaperone protein ClpB 1 |
|  |  |  | Vitvi17g01183 | Probable metal-nicotianamine transporter YSL7 |
|  |  |  | Vitvi17g00643 | LON peptidase N-terminal domain and RING finger protein 1 |
|  |  |  | Vitvi17g00291 | calmodulin binding |
|  |  |  | Vitvi17g00178 | Uncharacterized oxidoreductase ygbJ |
|  |  |  | Vitvi17g00936 | High-affinity nitrate transporter 3.2 |
|  |  |  | Vitvi17g00698 | Flavonoid 3'-monooxygenase |
|  |  |  | Vitvi17g00819 | Carbonic anhydrase chloroplastic |
|  |  |  | Vitvi17g00069 | Protein RUPTURED POLLEN GRAIN 1 |
|  |  |  | Vitvi17g00473 | Unknown Protein Function |
|  |  |  | Vitvi17g00804 | Serine/threonine-protein kinase HT1 |
|  |  |  | Vitvi17g00473 | Unknown Protein Function |
|  |  |  |  |  |
| SNPs | 19 | 21 | Vitvi19g01871 | Metallothionein-like protein type 3 |
|  |  |  | Vitvi19g01669 | Myb-related protein Zm1 |
|  |  |  | Vitvi19g00020 | Mitogen-activated protein kinase 9 |
|  |  |  | Vitvi19g00252 | 5'-AMP-activated protein kinase gamma subunit |
|  |  |  | Vitvi19g02032 | Unknown Protein Function |
|  |  |  | Vitvi19g01612 | Polygalacturonase |
|  |  |  | Vitvi19g00111 | Epidermis-specific secreted glycoprotein EP1 |
|  |  |  | Vitvi19g00270 | NAC domain-containing protein 72 |
|  |  |  | Vitvi19g00697 | Cellulose synthase-like protein E1 |
|  |  |  | Vitvi19g01824 | Probable LRR receptor-like serine/threonine-protein kinase RFK1 |
|  |  |  | Vitvi19g00257 | Protochlorophyllide reductase chloroplastic |
|  |  |  | Vitvi19g00700 | Cellulose synthase-like protein E1 |
|  |  |  | Vitvi19g02145 | Probable glutathione S-transferase parC |
|  |  |  | Vitvi19g00383 | Vacuolar H+-ATPase V0 sector subunit c'' |
|  |  |  | Vitvi19g01048 | Probable glutathione S-transferase parC |
|  |  |  | Vitvi19g00173 | Anthocyanidin 3-O-glucosyltransferase |
|  |  |  | Vitvi19g02157 | Potassium transporter 1 |
|  |  |  | Vitvi19g00623 | Zinc finger CCCH domain-containing protein 18 |
|  |  |  | Vitvi19g00601 | Pirin-like protein |
|  |  |  | Vitvi19g00041 | Unknown Protein Function |
|  |  |  | Vitvi19g00581 | Unknown Protein Function |

Marker= SNPs/InDels; Chr= chromosome; DE genes= differentially expressed genes; Gene_ID= Gene code based on reference genome for *V. vinifera* PN40024 12X.v1, and Vcost_v3 annotation [90].
